# Narciclasine attenuates diet-induced obesity by promoting oxidative metabolism in skeletal muscle

**DOI:** 10.1371/journal.pbio.1002597

**Published:** 2017-02-16

**Authors:** Sofi G. Julien, Sun-Yee Kim, Reinhard Brunmeir, Joanna R. Sinnakannu, Xiaojia Ge, Hongyu Li, Wei Ma, Jadegoud Yaligar, Bhanu Prakash KN, Sendhil S. Velan, Pia V. Röder, Qiongyi Zhang, Choon Kiat Sim, Jingyi Wu, Marta Garcia-Miralles, Mahmoud A. Pouladi, Wei Xie, Craig McFarlane, Weiping Han, Feng Xu

**Affiliations:** 1 Singapore Institute for Clinical Sciences, Agency for Science, Technology and Research (A*STAR), Singapore, Republic of Singapore; 2 Laboratory of Metabolic Medicine, Singapore Bioimaging Consortium, A*STAR, Singapore, Republic of Singapore; 3 Magnetic Resonance Spectroscopy and Metabolic Imaging Group, Singapore Bioimaging Consortium, A*STAR, Singapore, Republic of Singapore; 4 Institute of Molecular and Cell Biology, A*STAR, Singapore, Republic of Singapore; 5 Center for Stem Cell Biology and Regenerative Medicine, MOE Key Laboratory of Bioinformatics, THU-PKU Center for Life Sciences, School of Life Sciences, Tsinghua University, Beijing, China; 6 Translational Laboratory in Genetic Medicine, A*STAR, Singapore, Republic of Singapore; 7 Department of Medicine, Yong Loo Lin School of Medicine, National University of Singapore, Singapore, Republic of Singapore; University of California Los Angeles, UNITED STATES

## Abstract

Obesity develops when caloric intake exceeds metabolic needs. Promoting energy expenditure represents an attractive approach in the prevention of this fast-spreading epidemic. Here, we report a novel pharmacological strategy in which a natural compound, narciclasine (ncls), attenuates diet-induced obesity (DIO) in mice by promoting energy expenditure. Moreover, ncls promotes fat clearance from peripheral metabolic tissues, improves blood metabolic parameters in DIO mice, and protects these mice from the loss of voluntary physical activity. Further investigation suggested that ncls achieves these beneficial effects by promoting a shift from glycolytic to oxidative muscle fibers in the DIO mice thereby enhancing mitochondrial respiration and fatty acid oxidation (FAO) in the skeletal muscle. Moreover, ncls strongly activates AMPK signaling specifically in the skeletal muscle. The beneficial effects of ncls treatment in fat clearance and AMPK activation were faithfully reproduced in vitro in cultured murine and human primary myotubes. Mechanistically, ncls increases cellular cAMP concentration and ADP/ATP ratio, which further lead to the activation of AMPK signaling. Blocking AMPK signaling through a specific inhibitor significantly reduces FAO in myotubes. Finally, ncls also enhances mitochondrial membrane potential and reduces the formation of reactive oxygen species in cultured myotubes.

## Abstract 2

Narciclasine is a natural compound that attenuates diet-induced obesity in mice by promoting energy expenditure; it also induces a number of beneficial metabolic effects and activates AMPK signaling in skeletal muscle.

## Author summary

Obesity results from the imbalance of food intake and energy expenditure. Since the restriction of food intake is difficult and inefficient in maintaining long-term weight loss, enhancing energy expenditure is now an attractive approach in combating obesity. Here, we analysed the role in this process of a natural compound called narciclasine. We showed that narciclasine treatment reduces excess fat accumulation in peripheral metabolic tissues, improves blood metabolic parameters and insulin sensitivity in obese mice, and protects these mice from the loss of voluntary physical activity. Further investigation suggested that narciclasine enhances mitochondrial respiration and fatty acid consumption in the skeletal muscle. In addition, narciclasine strongly activates the AMP-activated protein kinase (AMPK) signaling, which is a central sensor of the cellular energy status and a key player in maintaining energy homeostasis, specifically in the skeletal muscle. Mechanistically, we found that narciclasine increases cAMP concentration and ADP/ATP ratio in muscle cells, which further lead to AMPK activation. Finally, we observed that narciclasine increases mitochondrial membrane potential and reduces the production of reactive oxygen species in muscle cells. Our findings suggest that narciclasine is a natural compound that attenuates diet-induced obesity in mice by promoting energy expenditure.

## Introduction

Obesity continues to spread in both industrial and developing countries, thus necessitating efficient therapeutic approaches to prevent this epidemic. In line with thermodynamics, any treatments for obesity must either reduce energy intake and/or increase energy expenditure [1–3]. Given that only 20% of individuals with dietary restrictions are able to maintain long-term weight loss [4], increasing energy expenditure is becoming an attractive approach to combat obesity. Towards this end, much effort has been focused on the search for novel pharmacological approaches that enhance energy expenditure to reduce adiposity and introduce beneficial metabolic effects in humans.

Obesity is associated with a number of metabolic dysfunctions including reduced fatty acid oxidation (FAO), increased lipid storage in peripheral tissues, and enhanced anaerobic glycolytic activity [2]. All of these negative impacts on metabolism can be counteracted by endurance exercise training. The beneficial metabolic adaptation upon endurance training mainly occurs in skeletal muscle, which is composed of myofibers that differ in their metabolic and contractile properties. These myofibers can be broadly divided into oxidative slow-twitch and glycolytic fast-twitch fibers [5], and conversion between the two fiber types occurs in response to contractile demands. For example, endurance exercise training promotes the formation of oxidative slow-twitch myofibers and enhances FAO in skeletal muscle, which contributes largely to the lipid oxidation among peripheral tissues involved in lipid homeostasis [6]. One of the major regulators of FAO that senses cellular energy status in skeletal muscle is the AMP-activated protein kinase (AMPK) [7, 8]. Activation of AMPK by phosphorylation at Thr172 of the catalytic subunit (α) promotes FAO by direct phosphorylation and inhibition of its downstream target acetyl-CoA carboxylase (ACC) [9]. During this process, AMPK activation also leads to the induction of transcriptional coactivator PGC1α [10, 11], which acts as a principle regulator that promotes the formation of oxidative slow-twitch myofibers [12], mitochondrial biogenesis [13, 14], electron transport chain function, and oxidative metabolism [15]. PGC1α exerts these functions by inducing an array of genes involved in oxidative metabolism and mitochondrial biogenesis, including mitochondrial transcription factor A (*Tfam*), cytochrome c oxidases (*Cox2* and *Cox4*), peroxisome proliferator-activated receptor alpha (*Ppara*), carnitine palmitoyltransferase 1B (*Cpt1b*), and many slow-twitch fiber markers [5, 12, 16, 17].

Several small molecules possess the capability of reducing fat accumulation and improving metabolic profiles by activating the AMPK signaling pathway. For example, resveratrol, a natural compound found in grapes, enhances mitochondrial biogenesis, metabolic rate, physical endurance, and attenuates high-fat diet (HFD)-induced obesity in rodents [18]. These beneficial effects are at least partially mediated through the activation of AMPK signaling, as in the AMPK-deficient mice, resveratrol failed to induce such metabolic improvements [19]. In addition, 5-Aminoimidazole-4-carboxamide ribonucleotide (AICAR), an AMP analog and hence an agonist of AMPK, promotes the expression of a panel of oxidative metabolism genes in quadricep muscles, increases running endurance, and reduces epididymal white fat mass [20]. Moreover, Metformin, the most widely used drug for type 2 diabetes worldwide, lowers lipid contents in liver, reduces blood glucose levels, and improves glucose tolerance through AMPK activation [21, 22]. In addition to its wide usage for diabetes, Metformin has also been proposed for polycystic ovary syndrome treatment and clinical trials for longevity and cancer prevention [23]. Indeed, cancer drug treatments often lead to alterations in energy metabolism of cancer patients [24], suggesting the potential application of these drugs in treating metabolic dysfunctions. In this study, we report that at physiological doses, narciclasine (ncls), a natural compound that displays marked anticancer activity in vitro [25, 26] and, in experimental preclinical models including melanoma and gliomas [27, 28], protects mice from diet-induced obesity (DIO) by enhancing energy expenditure without affecting food intake. We also showed that ncls reduces fat accumulation in peripheral metabolic tissues including liver, white adipose tissue (WAT), brown adipose tissue (BAT), and skeletal muscle, improves blood metabolic parameters, and protects HFD mice from the loss of voluntary physical activity. These beneficial metabolic effects were achieved by a shift from glycolytic to oxidative muscle fibers in the DIO mice leading to increased mitochondrial respiration and FAO likely through AMPK activation in skeletal muscle. Moreover, ncls promotes fat clearance and AMPK activation in a cell-autonomous manner in cultured murine and human primary myotubes. Mechanistically, ncls increases cAMP concentration and adenosine diphosphate (ADP)/ATP ratio in myotubes, leading further to the activation of AMPK signaling. In summary, our results indicated the therapeutic potential of ncls in combating obesity by increasing energy expenditure through enhancing FAO in skeletal muscle.

## Results

### Ncls attenuates DIO in mice without affecting growth

**Fig 1:**
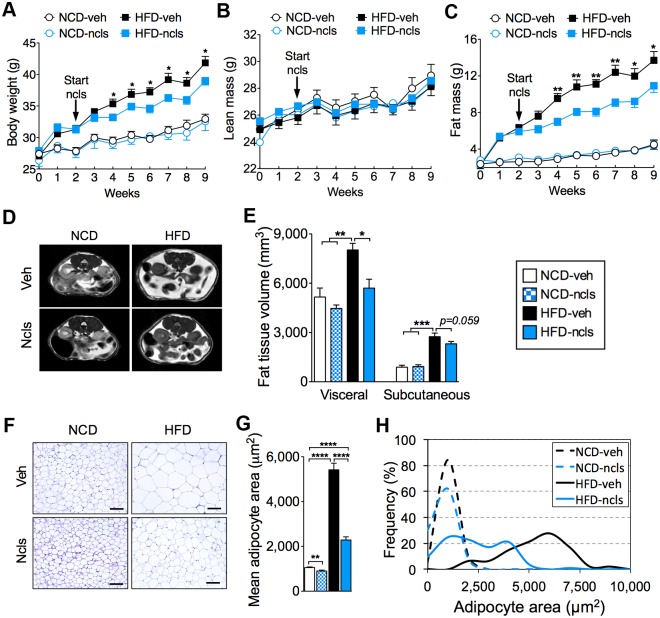
Ncls protects mice from DIO. (A) Total body weight, (B) lean mass, and (C) fat mass of ncls- or veh-treated mice fed on either an HFD or an NCD. (D) Representative MRI cross-sectional images showing the distribution of visceral and subcutaneous fat in the mice described in (A). (E) Quantitative analysis of the abdominal fat tissue volume (visceral and subcutaneous) by MRI. (F) Representative hematoxylin & eosin (H&E)-stained epididymal WAT sections from the mice described in (A). Scale bar, 100 μm. (G) Mean area of adipocytes from the H&E sections shown in (F). (H) Frequency distribution of adipocyte sizes from the H&E sections shown in (F). * *p* < 0.05, ** *p* < 0.01, *** *p* < 0.001, **** *p* < 0.0001. Underlying data and method of statistical analysis are provided in S1 Data.

To assess the effect of ncls on whole-body metabolism, we gavaged the mice weekly with either vehicle (veh) or ncls at 1 mg per kg of body weight for seven consecutive weeks, according to the previous in vivo studies [26, 28, 29]. When the mice were fed a normal chow diet (NCD), ncls exhibited no notable effect on body weight (Fig 1A), lean mass (Fig 1B), or fat mass (Fig 1C), suggesting that ncls did not affect normal growth when mice were under an energy-balanced condition. In contrast, ncls significantly reduced the body weight gain (Fig 1A) and fat mass accumulation (Fig 1C) after only 2 wk of treatment in mice intaking excess energy through HFD feeding. Nuclear magnetic resonance (NMR) analysis of body composition revealed that, in NCD mice, ncls treatment had no effect on the percentage of either fat mass or lean mass; however, in HFD mice, ncls significantly reduced the percentage of fat mass (S1A and S1B Fig). Further magnetic resonance imaging (MRI) analysis of fat distribution revealed that the reduction in fat mass by ncls treatment in HFD mice was mainly reflected by reductions in the visceral and, to a lesser extent, subcutaneous fat (Fig 1D and 1E). In accordance with the increased adiposity in HFD mice, histological assessment of epididymal WAT revealed that these mice contained larger adipocytes compared with the NCD mice (Fig 1F) [30, 31]. Notably, ncls treatment led to a substantial decrease in the adipocyte size in HFD mice (Fig 1F). The mean adipocyte area was also dramatically reduced by ncls treatment, from 5,419 ± 296 μm^2^ (HFD-veh mice) to 2,287 ± 141 μm^2^ (HFD-ncls mice, *p* < 0.0001) (Fig 1G). Concomitantly, there was a significant decrease in the population of large-sized adipocytes (Fig 1H).

**Fig 2:**
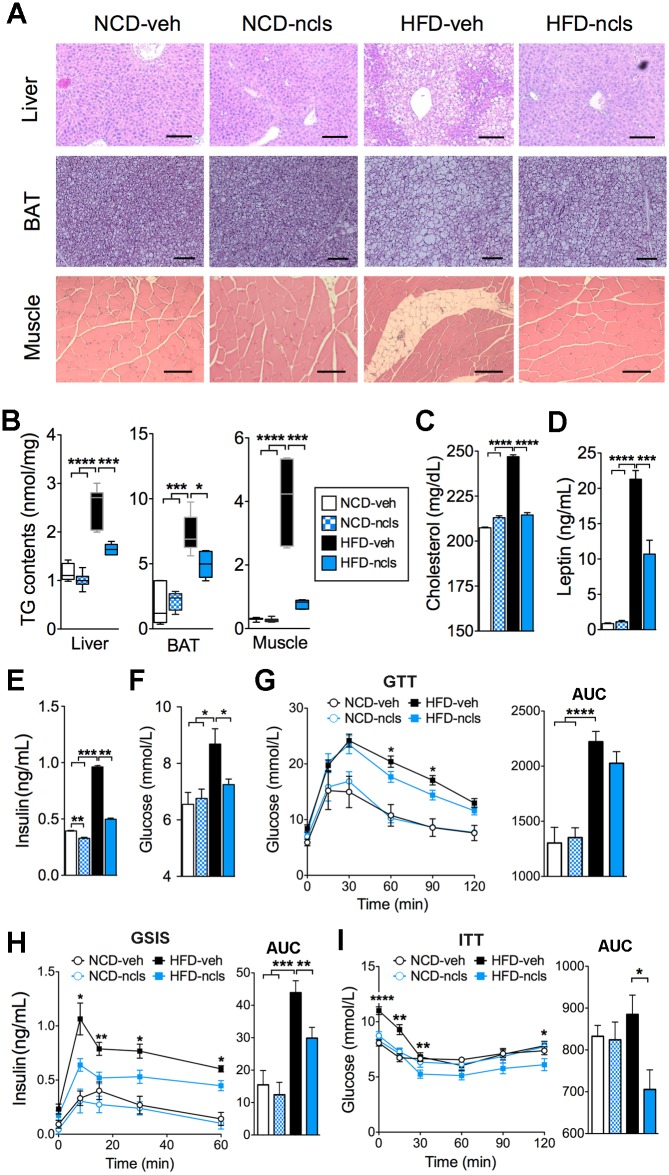
Ncls promotes fat clearance and improves blood metabolic parameters in HFD mice. (A) Representative H&E-stained sections of liver, BAT, and skeletal muscle (quadriceps) from mice treated for 7 wk with ncls or veh on either an HFD or an NCD. Scale bars, 100 μm. (B) TG contents in liver, BAT, and quadricep muscles of the mice described in (A). Blood metabolic parameters were determined after 6 wk of ncls or veh administration. (C) Cholesterol, (D) fasting plasma leptin, (E) fasting plasma insulin, and (F) fasting plasma glucose levels were shown as bar graphs. (G) Oral glucose tolerance test (GTT), (H) GSIS assay and (I) insulin tolerance test (ITT) were performed in mice with ncls or veh treatment on either an HFD or an NCD. Area under curve (AUC) was calculated and shown as bar graphs in the right panels. * *p* < 0.05, ** *p* < 0.01, *** *p* < 0.001, **** *p* < 0.0001. Underlying data and method of statistical analysis are provided in S1 Data.

In addition to the WAT, we also examined liver, BAT, and skeletal muscle for the impact of 7 wk’s ncls treatment on lipid accumulation. As shown by the hematoxylin & eosin (H&E) staining, hepatosteatosis, excess fat accumulation in BAT, and ectopic fat deposits in skeletal muscle, which are normally observed in HFD mice, were almost completely abolished by ncls treatment (Fig 2A). We also found significantly lower triglyceride (TG) content in the liver, BAT, and quadricep muscles (Fig 2B) of HFD-ncls mice compared with HFD-veh mice, indicating a beneficial effect of ncls on fat clearance in those organs. Moreover, HFD feeding leads to increased cholesterol, leptin, fasting insulin, and glucose levels in the blood, and these adverse effects were dramatically reduced by ncls treatment (Fig 2C–2F). In addition, ncls treatment slightly ameliorated the glucose intolerance in HFD mice (Fig 2G). Further analysis revealed that ncls treatment significantly reduced glucose-stimulated insulin secretion (GSIS) in HFD mice (Fig 2H). While insulin sensitivity was also improved by ncls in HFD mice (Fig 2I). Given that fat accumulation represents a net balance between energy expenditure and caloric intake, the marked decrease in adiposity in the HFD-ncls mice prompted us to examine the effects of ncls treatment on whole-body energy homeostasis in HFD mice using metabolic chambers.

### Ncls protects the HFD mice from reduced energy expenditure and physical activity

**Fig 3:**
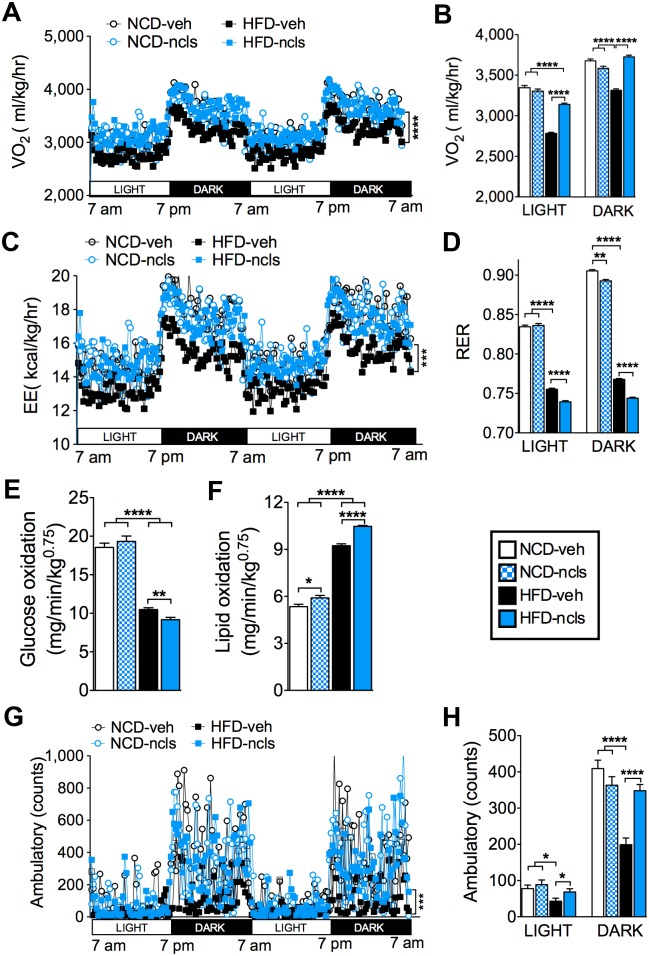
Ncls restores energy expenditure and physical activity of HFD mice to the levels of NCD mice. (A) Oxygen consumption (VO_2_) over a 48-h period of mice treated for 6 wk with ncls or veh on either an HFD or an NCD. (B) Averages of VO2 in light and dark cycles. (C) Energy expenditure (EE) of the mice described in (A) over a 48-h period. (D) Mean RER of the mice described in (A) during light and dark cycles. Whole body (E) glucose and (F) lipid oxidation rates of the mice described in (A). (G) Physical activity plots of the mice described in (A) over two light and dark cycles. (H) Mean ambulatory counts of the mice described in (A) during light and dark cycles. * *p* < 0.05, ** *p* < 0.01, *** *p* < 0.001, **** *p* < 0.0001. Underlying data and method of statistical analysis are provided in S1 Data.

To assess the effect of ncls on energy homeostasis, we monitored the metabolic parameters of ncls-treated mice by indirect calorimetry. As expected, HFD mice displayed significantly decreased oxygen consumption compared with NCD mice [32]. Strikingly, ncls administration restored oxygen consumption in HFD mice to the level of NCD mice (Fig 3A and 3B). This result was consistent with a significant increase in energy expenditure throughout both the light and dark cycles in HFD-ncls mice (Fig 3C). We next asked what the main energy source was in these HFD-ncls mice. To address this question, we calculated the respiratory exchange ratio (RER) based on oxygen consumption (Fig 3A) and carbon dioxide production (S2A Fig). As expected, HFD mice showed a lower RER compared with NCD mice (Fig 3D). Intriguingly, HFD-ncls mice exhibited an even lower RER (0.730 ± 0.001, *n* = 12, *p* < 0.0001) than HFD-veh mice (0.765 ± 0.001, *n* = 13) (Fig 3D and S2B Fig), indicating greater fat utilization upon ncls treatment (92% for HFD-ncls group versus 77% for the HFD-veh group, according to the energy equivalence of respiratory gas volumes [33]). To further investigate the substrate utilization, lipid and glucose oxidation rates were calculated according to the formulas of Ferrannini from the indirect calorimetric data [34, 35]. Our results showed that glucose and lipid utilization (Fig 3E and 3F) were inversely correlated between NCD- and HFD-fed groups, and ncls significantly increased lipid oxidation (Fig 3F) where it reduced glucose utilization (Fig 3E) in HFD mice. This is in agreement with the lower RER observed for HFD-ncls mice. In addition, the remarkable reduction in adiposity in HFD-ncls mice could not be accounted for by hypophagia, as the food intake and absolute caloric intake (S3A–S3C Fig) did not differ significantly between the ncls- and veh-treated HFD mice. To rule out the possibility that ncls treatment led to higher energy loss in feces and subsequent reduction of body weight and fat mass, we also examined feces production, fecal energy content, and total fecal energy output in all four groups of mice. And we found these three parameters did not differ much between ncls- and veh-treated groups (S3D–S3F Fig). These results provided evidences for equal energy absorption in ncls- and veh-treated mice and suggested that the increase in lipid oxidative metabolism was accounted for ncls’s function in reducing fat mass in HFD mice.

HFD feeding in mice leads to reduced voluntary physical activity mainly due to the increased adiposity. We found ncls treatment also protected the HFD mice from loss of physical activity during both the light (resting) and dark (active) cycles (Fig 3G and 3H). In contrast, ncls had no effect on physical activity in NCD mice (Fig 3G and 3H). These observations suggested that the protective effect of ncls on voluntary physical activity in HFD mice was secondary to the reduced adiposity in these mice (Fig 1). To examine the impact of ncls treatment on central nervous system, we performed a number of mouse behavior tests. In the limb clasping test, we noted no observable phenotype of limb clasping in HFD mice after ncls treatment (S4A Fig). This is in contrast to the observation made in the hyperactive *PGC-1α* null mice [36], which demonstrated frequent limb clasping. In the open field test, the HFD-ncls mice exhibited no difference in the time spent in the center and number of entries into the center as compared to HFD-veh mice (S4B Fig), indicating no anxiety phenotype in these mice. In the elevated plus maze test, again, we observed no difference between HFD-ncls and HFD-veh mice in the time spent in the open arms (S4C Fig). In summary, we observed no changes in anxiety after ncls treatment in HFD mice through these behavior tests. Moreover, ncls did not affect the normal circadian rhythms in terms of oxygen consumption (Fig 3A), carbon dioxide production (S2A Fig), or ambulatory movement (Fig 3G) in both NCD and HFD mice, again suggesting that ncls did not exert a major impact on the central nervous system.

### Ncls targets skeletal muscle to promote the expression of slow-twitch fiber marker genes

**Fig 4:**
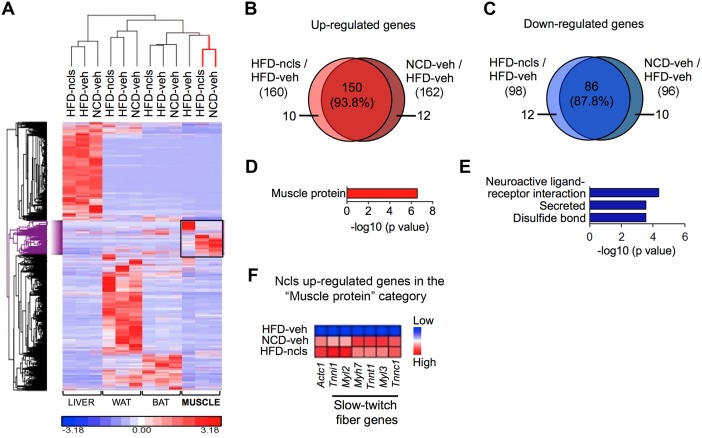
Ncls targets skeletal muscle to up-regulate signature genes of slow-twitch fibers in HFD mice. (A) Hierarchical clustering of the 1,532 DEGs (> 2-fold change) in liver, WAT, BAT, and quadricep muscles of NCD-veh, HFD-veh, and HFD-ncls mice. (B) Most of the ncls up-regulated genes in quadricep muscles of HFD mice were also highly expressed in NCD-veh mice. (C) Most of the ncls down-regulated genes in quadricep muscles of HFD mice were also expressed at a lower level in NCD-veh mice as compared with HFD-veh mice. The percentages of overlapping genes among the up- and down-regulated genes in HFD-ncls mice were indicated in (B) and (C). Gene ontology analyses of the overlapping (D) 150 up-regulated and (E) 86 down-regulated genes in quadricep muscles. (F) Expression patterns of the ncls up-regulated genes in the “muscle protein” category in NCD-veh, HFD-veh and HFD-ncls mice. Underlying data and method of statistical analysis are provided in S1 Data.

To determine the major in vivo target of ncls in promoting oxidative metabolism and inducing beneficial metabolic changes, we performed transcriptomic analyses using RNA-seq in WAT, BAT, liver, and skeletal muscle (quadriceps), the four major metabolic organs. Through hierarchical clustering analyses of the 1,532 differentially expressed genes (DEGs, > 2-fold change) across the four organs, we found that the gene expression patterns in WAT, BAT, and liver of HFD-ncls mice better resembled the patterns of HFD-veh mice than those of NCD-veh mice (Fig 4A), suggesting that ncls treatment did not have a profound effect on global gene expression in liver or adipose tissues. Intriguingly, in skeletal muscle, the gene expression profile of HFD-ncls mice better correlated with that of NCD-veh mice than that of HFD-veh mice (Fig 4A). This result demonstrated that ncls treatment largely restored the gene expression signature of HFD mice to that of NCD mice and indicated that skeletal muscle is a major in vivo target of ncls treatment. To decipher the molecular mechanism by which ncls treatment led to beneficial effects in HFD mice, we further analyzed the 258 DEGs identified by the hierarchical clustering in skeletal muscle (S1 Table). We found that 160 of these genes were up-regulated by ncls treatment in HFD mice, and 150 of them were also expressed at higher levels in NCD-veh mice than in HFD-veh mice (Fig 4B). Conversely, 98 genes were down-regulated by ncls treatment in HFD mice, among which 86 genes were also expressed at lower levels in NCD-veh mice (Fig 4C), reaffirming that ncls treatment largely restored the gene expression signature in skeletal muscle of HFD mice to that of NCD mice. We then performed functional annotation analysis using the web-based program DAVID to characterize the up- and down-regulated genes by ncls treatment. Of the 150 commonly up-regulated genes (Fig 4B), we found that “muscle protein” was the most enriched category (Fig 4D). The genes in this enriched category and their corresponding expression levels (fragments per kilobase of exon per million fragments mapped; FPKM) were provided in S2 and S3 Tables, respectively. We also found a few enriched categories from the 86 commonly down-regulated genes (Fig 4E). Strikingly, among the seven up-regulated genes in the “muscle protein” category (S2 Table), six of them were markers of oxidative slow-twitch muscle fibers (*Tnnt1*, *Tnni1*, *Tnnc1*, *Myl2*, *Myl3*, and *Myh7*, Fig 4F). This observation suggested that ncls treatment specifies the gene expression signature of oxidative muscle fibers and promotes oxidative metabolism in skeletal muscle. We also compared the transcriptomes of skeletal muscle from NCD-veh and NCD-ncls mice, and we observed a very high correlation in their gene expression profiles (Pearson correlation coefficient, R = 0.999) (S5 Fig). These results suggest that ncls only protects skeletal muscle in HFD mice from decreased oxidative gene expression and damage to oxidative capacity, but has little effect on muscle under normal growth conditions (NCD mice).

### Ncls enhances oxidative metabolism in the skeletal muscle of HFD mice

**Fig 5:**
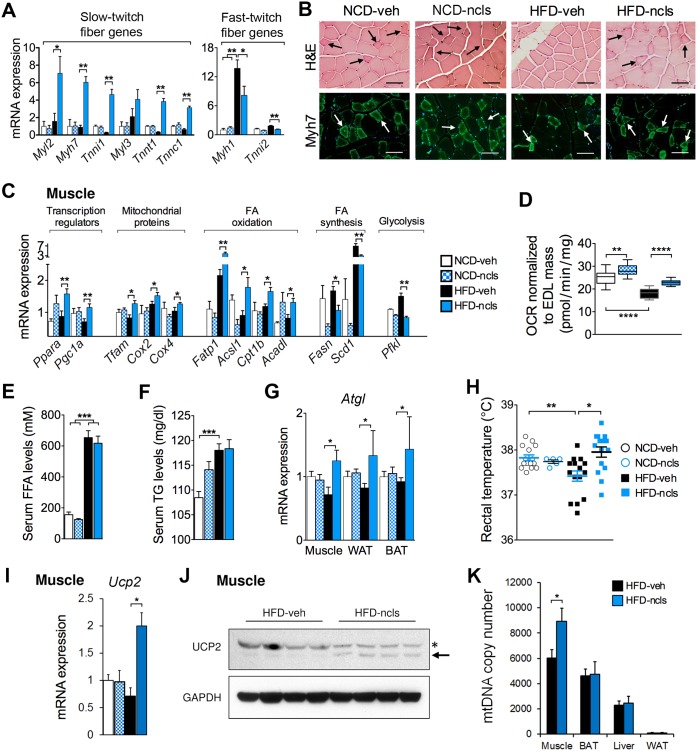
Ncls promotes slow-twitch fiber formation and oxidative metabolism in the skeletal muscle of HFD mice. (A) Relative mRNA expression of slow-twitch and fast-twitch fiber marker genes in quadricep muscles in ncls- or veh-treated mice on either an HFD or an NCD. The values of the NCD-veh mice were arbitrarily set as one. (B) Representative H&E staining (top panels) and Myh7 immunostaining (lower panels) of quadricep muscles from the mice described in (A). Oxidative slow-twitch fibers are indicated by arrows, and they are smaller in size and redder in color as compared to glycolytic fast-twitch fibers. Myh7-positive fibers are also indicated by arrows. Scale bar, 50 μm. (C) qRT-PCR analysis of the selected metabolic genes in quadricep muscles from all groups of mice. (D) Mitochondrial respiration rate of intact EDL muscle assayed on a Seahorse extracellular flux analyzer from all four groups of mice. Quantification of serum free fatty acid (FFA) (E) and TG (F) in ncls- or veh-treated mice. (G) Relative mRNA expression of *Atgl* gene in quadricep muscles, WAT, and BAT. (H) Core body temperatures of the mice described in (A). (I) *Ucp2* mRNA and (J) UCP2 protein levels in quadriceps muscle of veh- and ncls-treated mice were evaluated by qRT-PCR and western blotting. The asterisk denotes an unspecific band in the western blot, and the UCP2 band is indicated by an arrow. Glyceraldehyde 3-phosphate dehydrogenase (GAPDH) was included as a loading control. (K) Mitochondrial DNA copy numbers in muscle, BAT, liver, and WAT were determined by qPCR and shown as bar graphs. * *p* < 0.05, ** *p* < 0.01, *** *p* < 0.001, **** *p* < 0.0001. Underlying data and method of statistical analysis are provided in S1 Data.

To validate the RNA-seq data, we used quantitative reverse transcription polymerase chain reaction (qRT-PCR) to examine the expression of the signature genes of slow-twitch muscle fibers in quadriceps muscle from all groups of mice. Notably, ncls significantly up-regulated a number of slow-twitch fiber markers such as *Myl2*, *Myh7*, *Tnni1*, *Myl3*, *Tnnt1*, and *Tnnc1*, and down-regulated the signature genes of fast-twitch fibers, including *Myh1* and *Tnni2*, in the quadricep muscles from HFD mice (Fig 5A). These results suggested that ncls treatment induced a shift from glycolytic fast-twitch fibers toward oxidative slow-twitch fibers in quadricep muscles of HFD mice. Regarding this observation, we investigated the effects of ncls on muscle function. For muscle function, we first measured the grip strength of the mice from all four groups. We found that HFD feeding led to reduced skeletal muscle strength, and ncls partially restored muscle strength in HFD mice to the level of NCD mice (S6A Fig). We then examined the mass of extensor digitorum longus (EDL), soleus, tibialis anterior (TA), gastrocnemius (Gastr.) and quadricep muscles and found no difference in individual muscle mass between the ncls-treated and veh-treated groups of mice (S6B–S6F Fig).

To further confirm the shift from glycolytic fast-twitch fibers toward oxidative slow-twitch fibers, H&E cross-sections of quadricep muscles from HFD-ncls mice were compared with sections from NCD-veh, NCD-ncls, and HFD-veh mice. As shown in Fig 5B, ncls treatment led to a significant increase in the number of oxidative slow-twitch fibers, which are smaller in size and redder in color as compared to glycolytic fast-twitch fibers in quadriceps muscle of HFD mice. Immunostaining of the slow-twitch fiber marker Myh7 further confirmed this phenotype as a significant increase in Myh7-positive fibers was observed in the quadricep muscles from ncls-treated HFD mice (Fig 5B). Moreover, muscle fiber-typing experiments further showed that both the oxidative myosin heavy chain (MHC) I and MHC IIa fibers increased after ncls treatment in HFD mice, while the glycolytic MHC IIb fiber decreased (S7B and S7C Fig). The changes in muscle fiber types were in agreement with decreased muscle fiber cross-sectional areas (CSAs) in HFD-ncls mice (S7A Fig), as MHC I and MHC IIa fibers are smaller in CSAs when compared to MHC IIb fibers [37–39]. To clarify whether the fiber-type switch caused by ncls treatment was accompanied by changes in energy metabolism pathways, we reanalyzed the RNA-seq data using a 1.4-fold change cutoff to allow the identification of less significantly changed DEGs in skeletal muscle. We found that, indeed, “metabolic process” was identified as the most enriched category from the genes affected by ncls treatment (S8A Fig). According to the RNA-seq (S8B Fig) and qRT-PCR data in WAT, BAT, liver, heart (S9 Fig), and muscle (Fig 5C), genes involved in mitochondrial activity (*Tfam*, *Cox2* and *Cox4*) and FAO (fatty acid transport protein 1 (*Fatp1*), acyl-CoA synthetase long-chain family member 1 (*Acsl1*), acyl-CoA dehydrogenase, long chain (*Acadl*), and *Cpt1b*) were markedly up-regulated by ncls, specifically in muscle of HFD mice, whereas genes involved in fatty acid synthesis (stearoyl-CoA desaturase (*Scd1*) and fatty acid synthase (*Fasn*)) were significantly down-regulated by ncls only in muscle. Notably, PGC1α and PPARα, two transcription regulators that specify the gene expression program of oxidative metabolism in skeletal muscle, were also up-regulated by ncls in HFD mice (*p* < 0.01, *n* = 12) (Fig 5C and S8B Fig). These results were in line with the lower RER in HFD-ncls mice shown earlier in Fig 3D, confirming that fatty acids are the main fuel source for mitochondria in muscle of HFD-ncls mice. Given that there was a switch from glycolytic fast-twitch fibers to oxidative slow-twitch fibers in HFD mice upon ncls treatment (Fig 5A and 5B, S7B and S7C Fig), it was not surprising to observe a decreased expression of glycolytic genes such as phosphofructokinase (*Pfkl*) in HFD-ncls mice (Fig 5C). To directly address the question of whether ncls enhances mitochondrial respiration in the skeletal muscle of mice, we measured the mitochondrial oxygen consumption rates (OCRs) in intact EDL muscles isolated from all groups of mice ex vivo using the Seahorse extracellular flux analyzer. We observed significantly higher mitochondrial respiration in EDL muscles from HFD-ncls mice than those from HFD-veh mice (Fig 5D), confirming an increased oxidative metabolism in the skeletal muscle of HFD-ncls mice.

Excess energy is mainly stored in adipose tissues in the form of TGs; when needed, TGs are mobilized through lipolysis to supply free fatty acid (FFA) to skeletal muscle. We showed that ncls promoted the formation of oxidative muscle fibers and, presumably, there would be an increase in FFA demands in skeletal muscle. Indeed, we observed similarly high levels of serum FFA (Fig 5E) and TG (Fig 5F) in HFD-ncls mice as compared to HFD-veh mice, despite the reduced adiposity in these mice. In addition, these changes were accompanied by increased expression of the key lipolytic enzyme, *Atgl* [40] (also known as *Pnpla2*), in both WAT and BAT upon ncls treatment (Fig 5G). Moreover, *Atgl* was also significantly induced by ncls in the quadricep muscles of HFD mice (Fig 5G). These results were in agreement with the enhanced fat clearance from these tissues (Figs 1 and 2A). In summary, we conclude that ncls enhances oxidative metabolism in skeletal muscle, and the main fuel source is FFA derived from lipolysis in peripheral tissues.

Next, we asked where the extra energy produced from enhanced oxidative metabolism in HFD-ncls mice was spent. One answer is that part of the energy was used for increased physical activities (Fig 3G and 3H) in these mice. In addition, we also asked whether HFD-ncls mice spent more energy on nonshivering thermogenesis than HFD-veh mice. In this regard, we measured the core body temperatures of all groups of mice and, indeed, we found HFD-ncls mice have higher rectal temperatures than HFD-veh mice (Fig 5H), suggesting enhanced thermogenesis. Moreover, we detected increased *Ucp2* mRNA and UCP2 protein levels in the skeletal muscle from HFD-ncls mice by qRT-PCR and western blotting (Fig 5I and 5J). In parallel, we also found an increase in mitochondrial DNA copy number, specifically in the skeletal muscle of HFD mice after ncls treatment, indicating enhanced mitochondrial biogenesis in the muscle, but not in the BAT, liver, or WAT (Fig 5K). Together, we conclude that the increased energy supply from enhanced oxidative metabolism in HFD-ncls mice was utilized for both physical activity as well as nonshivering thermogenesis in the skeletal muscle.

### Ncls enhances mitochondrial respiration and FAO in murine and human primary myotubes

**Fig 6:**
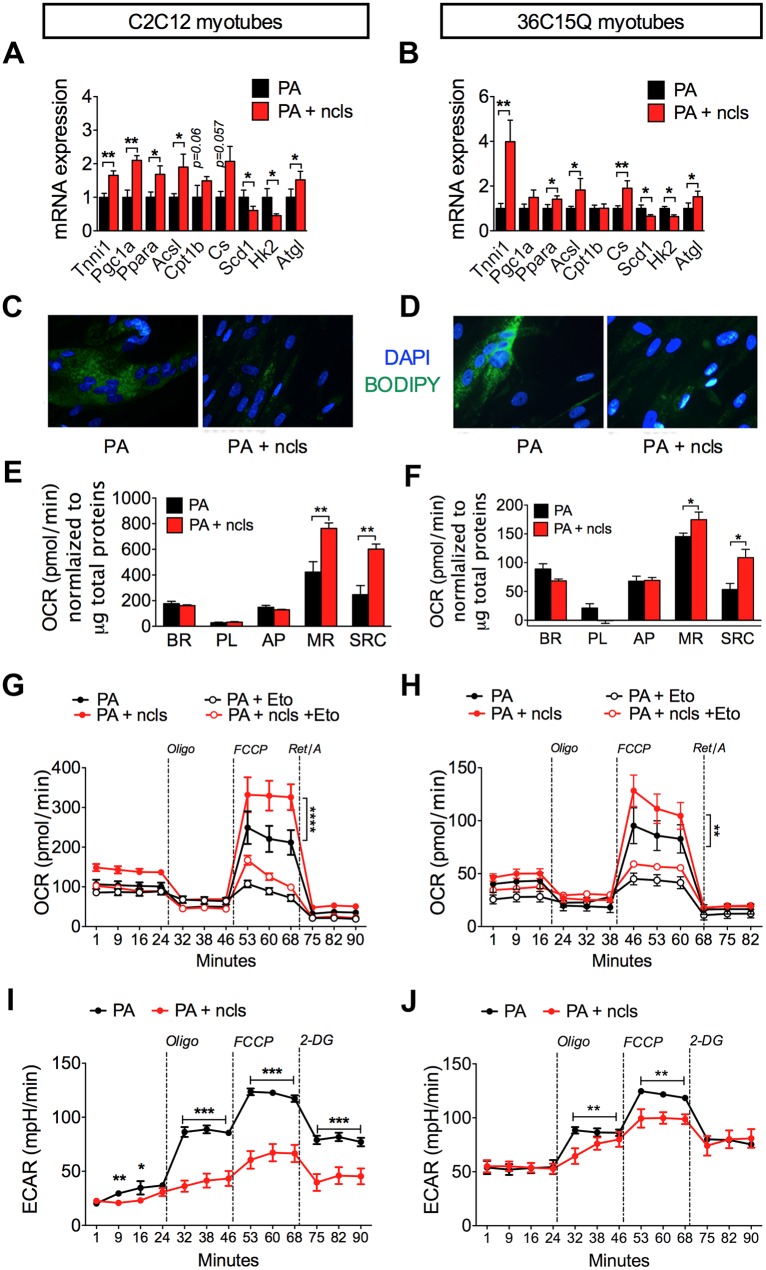
Ncls enhances mitochondrial respiration and FAO in both murine and primary human myotubes. Relative mRNA expression of selected metabolic genes in (A) murine myotubes (C2C12) and (B) primary human myotubes (36C15Q) after exposure to 0.1 mM PA ± 20 nM ncls for 48 h. The values of the PA-treated myotubes were arbitrarily set as one. (C) C2C12 and (D) 36C15Q myotubes were stained with BODIPY (green) for lipid droplets and DAPI (blue) for nuclei (40x magnification). Mitochondrial respiration was evaluated with a Seahorse extracellular flux analyzer. The OCR output for the basal rate (BR), proton leak (PL), ATP production (AP), MR, and spare respiratory capacity (SRC) of ncls-treated (E) C2C12 and (F) 36C15Q myotubes were shown as bar graphs. The OCR was normalized to the total protein per well. FAO profiles of (G) C2C12 and (H) 36C15Q myotubes were determined by a Seahorse extracellular flux analyzer. Myotubes were incubated in substrate-limited medium overnight to prime the myotubes for utilization of exogenous fatty acids and assayed on the following day with 0.125 mM PA. Etomoxir (Eto, 40 μM) was used to inhibit FAO and to confirm the assay specificity. Vertical dashed lines indicate the time points of oligomycin (Oligo, 1 μM), FCCP (1.6 μM), and Rotenone/Antimycin A (Ret/A, 1 μM) injection. The ECAR of (I) C2C12 and (J) 36C15Q myotubes were determined by a Seahorse analyzer after treatment with 0.1 mM PA ± 20 nM ncls for 48 h. Vertical dashed lines indicate the time points of oligomycin (1.5 μM), FCCP (1.5 μM), and 2-deoxyglucose (2-DG, 100 mM) injection. * *p* < 0.05, ** *p* < 0.01, *** *p* < 0.001, **** *p* < 0.0001. Underlying data and method of statistical analysis are provided in S1 Data.

To further validate the effects of ncls treatment on skeletal muscle, we used in vitro-differentiated myotubes from murine C2C12 cells and human 36C15Q primary myoblasts [41, 42]. To mimic the HFD feeding used in the animal study, we treated differentiated myotubes with 0.1 mM palmitate (PA) for 48 h in either the presence or absence of 20 nM ncls. As shown in Fig 6A and 6B, ncls treatment induced the expression of an array of genes involved in oxidative metabolism such as *Tnni1*, *Pgc1a*, *Ppara*, *Acsl*, and *Cpt1b* in both cell lines. Intriguingly, citrate synthase (*Cs*), which is significantly activated in well-trained athletes [43], was also up-regulated by ncls treatment. Consistent with the in vivo results, expression of the lipolytic gene *Atgl* increased in myotubes treated with ncls. In contrast, genes involved in fatty acid storage (*Scd1*) or glucose metabolism (*Hk2*) were down-regulated (Fig 6A and 6B). These changes in gene expression were mirrored by a marked decrease in the lipid content [44] in ncls-treated C2C12 and human primary myotubes, as determined by BODIPY staining (Fig 6C and 6D). To ask whether the expression changes in metabolic genes were associated with changes in mitochondrial oxidative metabolism, we used the Seahorse extracellular flux analyzer to monitor mitochondrial functions [45, 46]. We found that the mitochondrial respiration was indeed enhanced by ncls treatment and the stimulation of the mitochondrial OCR in ncls-treated myotubes following carbonilcyanide p-triflouromethoxyphenylhydrazone (FCCP) injection (maximal respiration; MR) was the most obvious difference between PA- and PA-ncls—treated myotubes (Fig 6E and 6F). To further confirm that the enhanced mitochondrial respiration by ncls treatment was due to an increase of lipid oxidation as previously observed in HFD-ncls mice (Figs 3F and 5D), we evaluated FAO in myotubes using the Seahorse analyzer. As shown in Fig 6G and 6H, ncls significantly enhanced FAO in both murine and human myotubes. These effects were blocked by the treatment of etomoxir, a CPT1 inhibitor, confirming that the increased oxygen consumption was indeed derived from the oxidation of exogenous fatty acids. On the other hand, we also observed that ncls significantly reduced the extracellular acidification rate (ECAR) in C2C12 and 36C15Q myotubes (Fig 6I and 6J), indicating decreased glycolysis in these cells. In myotubes without PA treatment, ncls also increased the expression of some of the oxidative metabolism genes (S10A and S10B Fig), as well as mitochondrial respiration (S10C–S10F Fig), albeit to a much lesser extent as compared to PA-treated myotubes (Fig 6), especially in human 36C15Q myotubes. We interpret these results to mean PA treatment damages the metabolic function of myotubes and ncls significantly protects the cells from these negative impacts, while in healthy myotubes, these protective effects are less obvious. This is also in agreement with the effects of ncls treatment in animal models, which showed that ncls had less obvious effects in NCD mice but significantly protected HFD mice from a number of metabolic dysfunctions. Taken together, these data demonstrated that ncls promotes FAO and reduces glycolytic capacity in differentiated myotubes in vitro, as previously suggested by the gene expression changes in Figs 5C, 6A and 6B.

### Ncls activates AMPK signaling pathway in vitro and in vivo

**Fig 7:**
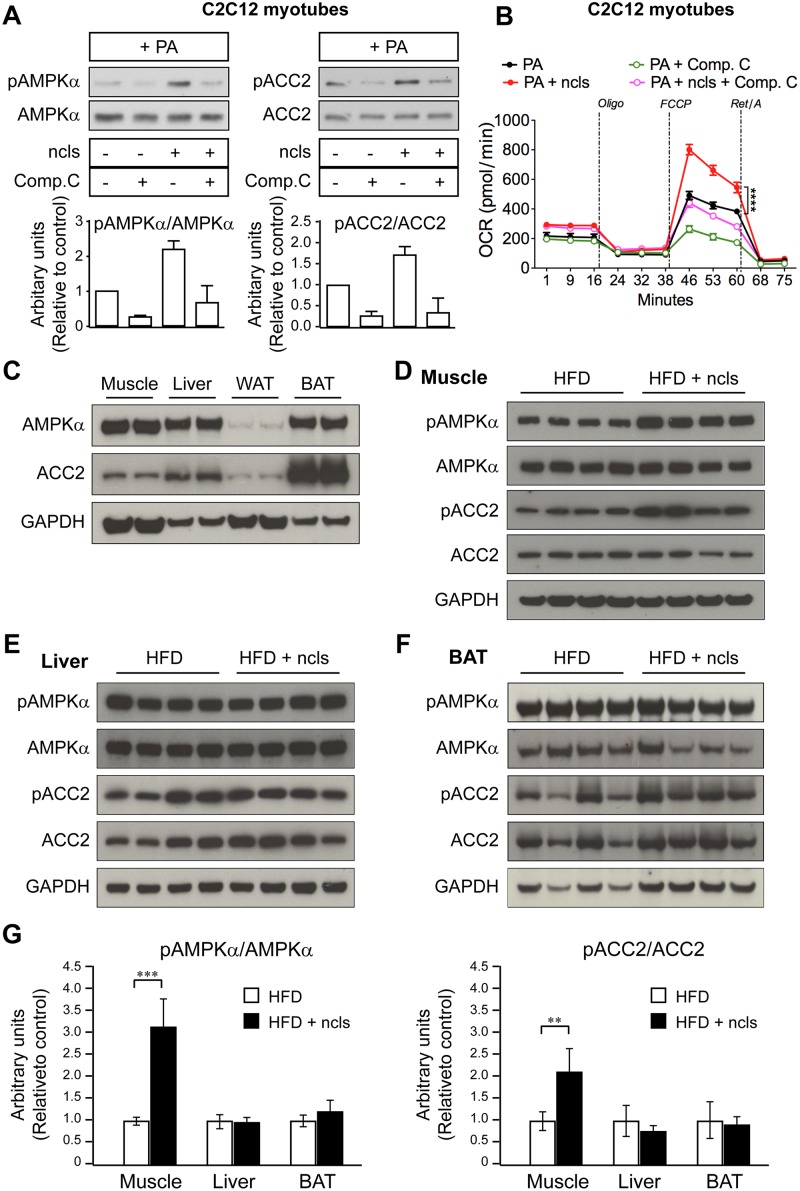
Ncls activates the AMPK signaling pathway in vitro and in vivo. Ncls treatment led to (A) increased phospho-AMPKα (pAMPKα), increased phospho-ACC2 (pACC2), and (B) enhanced FAO in PA-treated C2C12 myotubes. These effects were significantly reduced by treatment of Compound C (Comp. C), a specific AMPK inhibitor. Western blots of total AMPKα and ACC2 (A) were used as controls for equal loading. Ratios of phospho- to total- AMPKα and ACC2 were shown as bar graphs below the representative immunoblotting images of two independent experiments performed in duplicates. Vertical dashed lines indicate the time points of oligomycin (Oligo, 1 μM), FCCP (1.6 μM), and Rotenone/Antimycin A (Ret/A, 1 μM) injection (B). OCRs were determined by a Seahorse extracellular flux analyzer. **** *p* < 0.0001. (C) Protein levels of AMPKα and ACC2 were evaluated by western blotting in quadricep muscles, liver, WAT, and BAT from HFD-veh mice. GAPDH was included as a loading control. Ncls treatment led to increased pAMPKα and pACC2 specifically in (D) muscle, but not in (E) liver or (F) BAT from HFD mice. (G) Ratios of phospho- to total- AMPKα and ACC2 were shown as bar graphs. ** *p* < 0.01, *** *p* < 0.001. Underlying data and method of statistical analysis are provided in S1 Data.

It is well established that the activation of AMPK signaling pathway leads to increased FAO in skeletal muscle by phosphorylating and inhibiting ACC2, thereby promoting fatty acid uptake by mitochondria [3, 47]. To ask whether ncls acts through the AMPK pathway to stimulate FAO, we examined AMPKα and ACC2 phosphorylation levels in PA-treated C2C12 myotubes with or without ncls treatment. We observed that ncls stimulated phosphorylation of AMPKα at Thr172 and its downstream target ACC2 (Fig 7A), demonstrating the activation of the AMPK signaling pathway. AMPK activation by ncls further led to enhanced FAO as examined by a Seahorse extracellular flux analyzer (Fig 7B). Preincubation of myotubes with Compound C, a specific AMPK inhibitor, significantly reduced AMPKα phosphorylation, ACC2 phosphorylation (Fig 7A), and ncls-induced FAO (Fig 7B). These results demonstrated that ncls promotes FAO, at least partially, through AMPK activation in cultured myotubes in vitro. We also noted that Compound C treatment did not fully block the ncls-induced FAO (Fig 7B). This observation suggested that ncls may enhance mitochondrial respiration through both AMPK dependent and independent mechanisms. In C2C12 myotubes not subjected to PA treatment, ncls still had an effect in activing AMPK signaling (S11A Fig), albeit to a lesser extent as compared to PA-treated C2C12 myotubes (Fig 7A).

We next examined the effects of ncls on AMPK signaling in vivo. We first evaluated the protein expression of AMPKα and ACC2 in skeletal muscle, liver, WAT, and BAT, the four major metabolic organs using western blotting. As shown in Fig 7C, AMPKα and ACC2 were robustly expressed in muscle, liver, and BAT. In contrast, they were expressed at negligible levels in WAT. We thus focused the subsequent analyses on muscle, liver, and BAT. In agreement with the elevated expression of FAO genes in quadricep muscles of HFD-ncls mice (Fig 5C), we observed increased phosphorylation of AMPKα and ACC2 in the same tissue from ncls-treated HFD mice (Fig 7D and 7G). This phenotype was not observed in liver or BAT (Fig 7E–7G), suggesting that ncls specifically activated AMPK signaling in skeletal muscle. We also examined the effects of ncls on AMPK activation in the quadricep muscles of NCD mice. Results revealed no obvious differences in phospho-AMPKα (pAMPKα) and phospho-ACC2 (pACC2) between NCD-veh and NCD-ncls groups of mice (S11B Fig). In summary, ncls activates AMPK signaling pathway in vitro in C2C12 myotubes and in vivo in skeletal muscle of HFD mice.

### Ncls enhances mitochondrial membrane potential, increases cAMP concentration and ADP/ATP ratio, and reduces reactive oxygen species production

**Fig 8:**
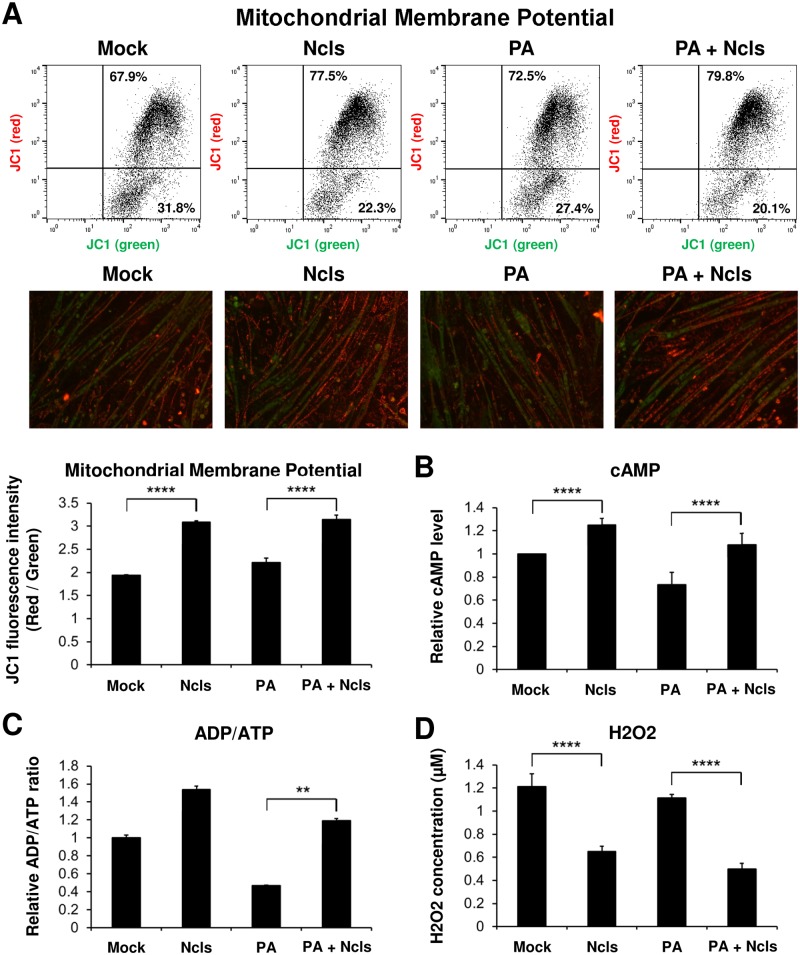
Biochemical basis for AMPK activation by ncls and the impact of ncls treatment on mitochondrial functions. (A) Ncls treatment led to increased MMP in C2C12 myotubes with or without PA treatment. The membrane-permeant JC-1 dye was used to monitor MMP in myotubes subjected to indicated treatments. Both flow cytometry and fluorescence microscopy results were shown for comparison. (B) cAMP concentration and (C) ADP/ATP ratio were examined in both ncls- and veh-treated C2C12 myotubes with or without PA treatment. (D) Hydrogen peroxide (H2O2) concentration was determined by an Amplex Red based assay kit in ncls- and veh-treated C2C12 myotubes with or without PA treatment. Data are presented as mean ± standard error of the mean (SEM). * *p* < 0.05, ** *p* < 0.01, **** *p* < 0.0001. Underlying data and method of statistical analysis are provided in S1 Data.

It is known that AMPK activation leads to the up-regulation of mitochondrial function [48]. After demonstrating the effect of ncls treatment on AMPK activation, we next investigated the impact of ncls treatment on mitochondrial transmembrane potential. Mitochondrial membrane potential (MMP) is an important parameter of mitochondrial function, and it indicates the status of cell health. Using the membrane-permeant JC-1 dye, we monitored MMP in both ncls- and veh-treated C2C12 myotubes. We found that ncls treatment strongly increased the concentration of JC-1 dye in mitochondria, which led to the formation of J-aggregates (Red color) in C2C12 myotubes as assayed by both flow cytometry and fluorescence microscopy (Fig 8A), suggesting enhanced MMP after ncls treatment. The increases in MMP upon ncls treatment were observed in both PA-treated and untreated myotubes, suggesting ncls improves MMP status in both settings. As an attempt to decipher the biochemical mechanism underlying AMPK activation by ncls, we also measured the cellular concentration of cAMP and the ADP/ATP ratio in C2C12 myotubes [49–54]. As shown in Fig 8B and 8C, ncls treatment up-regulated both cAMP concentration and ADP/ATP ratio in C2C12 myotubes with or without PA treatment. And a larger increase in cAMP concentration and ADP/ATP ratio was observed in PA-treated C2C12 myotubes after ncls treatment. This pattern is consistent with the observation of stronger AMPK activation in PA-treated C2C12 myotubes (Fig 7A) as compared to the untreated myotubes (S11A Fig). Together, these results provided biochemical basis for the effect of ncls on AMPK activation. In addition, we also examined the effect of ncls treatment on reactive oxygen species (ROS) production. In this assay, hydrogen peroxide concentration was determined in C2C12 myotubes with or without ncls treatment. We found that ncls significantly reduced the H2O2 concentration in both PA-treated and untreated C2C12 myotubes (Fig 8D), suggesting a prominent role for ncls in regulating ROS production.

## Discussion

Obesity is caused by excess caloric intake and/or insufficient energy usage. Therefore, enhancing energy expenditure, especially through pharmacological approaches, could have a major impact in combating obesity and its associated metabolic dysfunctions. In this study, we show that ncls treatment introduces a number of protective effects in mice against the metabolic damages caused by HFD feeding. These beneficial effects include ameliorated adiposity, increased energy expenditure, enhanced FAO, increased fat clearance from peripheral tissues, improved blood metabolic parameters, insulin sensitivity, and voluntary physical activity. Through transcriptomic analysis, we found that the global gene expression pattern of HFD mice reverted back to that of NCD mice only in skeletal muscle but not in the other three major metabolic organs WAT, BAT, and liver after ncls treatment, suggesting skeletal muscle is a major in vivo target of ncls. In quadricep muscles, ncls promoted the expression of PGC1α, the shift from glycolytic fast-twitch fibers to oxidative slow-twitch fibers, and the oxidation of FA. By increasing fatty acid consumption in skeletal muscle, ncls triggered a series of gene expression changes in various metabolic organs. These changes include increased expression of the key lipolytic enzyme *Atgl* in WAT, BAT, and skeletal muscle, along with decreased expression of the fatty acid storage genes *Scd1* and *Fasn* in skeletal muscle. Moreover, the enhanced oxidative metabolism and gene expression changes that we observed in skeletal muscle in vivo were largely reproduced in differentiated myotubes of both murine and human origin in vitro, indicating these effects were cell-autonomous. Based on these observations, we conclude that ncls enhances fatty acid utilization in skeletal muscle, thereby driving the mobilization of fatty acid and TG from other metabolic organs, and thus attenuates DIO in the DIO animal model.

Mechanistically, we found that ncls promotes oxidative metabolism in muscle likely through the activation of the AMPK signaling pathway. It is well documented that in skeletal muscle, the activation of the AMPK pathway by phosphorylation of AMPKα Thr172 leads to phosphorylation of ACC2, which inhibits the activity of this enzyme and reduces the conversion of acetyl-CoA to malonyl-CoA [8, 9]. Given malonyl-CoA is a potent inhibitor of CPT-1, a key enzyme for mitochondrial fatty acid uptake, the reduced level of malonyl-CoA leads to increased fatty acid uptake and oxidation in mitochondria [55]. Thus, activation of the AMPK pathway as manifested by increased pAMPKα and pACC2 promotes oxidative metabolism. AMPK activation introduces a number of metabolic effects that lead to increased ATP production and decreased ATP consumption in various tissues [7]. For example, activation of AMPK inhibits fatty acid synthesis in both liver and adipose tissues, while in skeletal muscle it enhances mitochondrial biogenesis and fatty acid uptake and oxidation. The stimulation of FAO is central to the beneficial effects of AMPK activation on reducing fat accumulation in peripheral tissues [7]. Consistent with this idea, the AMPKα2 knockout mice exhibit increased adiposity as compared to wild-type animals when given an HFD [56], while the ACC2 knockout, which mimics the constitutive activation of AMPK, leads to increased FAO in skeletal muscle and leanness [57]. Ncls administration enhances the expression of a panel of metabolic genes involved in mitochondrial biogenesis (especially *Pgc1a*) and FAO (Fig 5C) in skeletal muscle of HFD mice. As a result, we observed increased mitochondrial biogenesis (Fig 5K) in quadricep muscles of HFD-ncls mice. Ncls also prevents fat accumulation in peripheral tissues and improves blood metabolic profiles including fasting glucose and insulin levels. These beneficial effects are reminiscent of those introduced by AMPK activation, suggesting the involvement of AMPK signaling in ncls function. We thus investigated along this line and found that ncls treatment indeed led to significant increases in pAMPKα and pACC2 in C2C12 myotubes (Fig 7A), indicating strong activation of the AMPK pathway. This effect was significantly reduced by the treatment of Compound C, an AMPK inhibitor. Moreover, consistent with the observations in vivo (Fig 5D), ncls treatment enhanced FAO in myotubes as determined by the Seahorse extracellular flux analyzer (Fig 6G and 6H). This effect was at least partially dependent on the AMPK signaling, as Compound C treatment largely compromised the ncls-induced FAO (Fig 7B). In vivo studies further revealed elevated levels of pAMPKα and pACC2, hence increased AMPK activation, in the skeletal muscle of HFD mice, but not in BAT or the liver, where AMPKα and ACC2 are also robustly expressed (Fig 7). Previous studies showed that AMPK can be phosphorylated by upstream kinases including liver kinase B1 (LKB1) and Ca(2+)/calmodulin-dependent protein kinase kinase (CaMKK) [7]. Moreover, LKB1 has been suggested to be constitutively active, which indicates the existence of alternative regulatory mechanisms involved in AMPK phosphorylation. Whether ncls directly targets these kinases to activate AMPK or acts through alternative mechanisms warrants further investigation. Regarding the biochemical basis for ncls-mediated activation of AMPK signaling, we found that ncls treatment significantly up-regulated cAMP and increased ADP/ATP ratio in C2C12 myotubes (Fig 8B and 8C). These observations suggested that ncls may activate AMPK signaling via a similar mechanism as resveratrol [49]. Based on the data obtained through this study, we would like to propose the following model of ncls action: ncls treatment leads to increases in cAMP concentration and ADP:ATP ratio (Fig 8B and 8C), which promotes AMPK activation (Fig 7 and S11A Fig). The activation of AMPK signaling enhances PGC1α expression (Figs 5C and 6A and S10A Fig). As the principle regulator of mitochondrial biogenesis, activated PGC1α increases mitochondrial mass (Fig 5K), which then increases MMP as measured by JC1 staining (Fig 8A). In summary, we demonstrated a positive impact of ncls on the whole-body energy metabolism in HFD mice and have further unraveled a major signaling pathway through which ncls enhances oxidative metabolism. These findings propose ncls administration as a potential pharmacological strategy for obesity treatment.

## Materials and methods

### Animal studies

All animal procedures were performed according to the approved protocol (IACUC#130829) from Institutional Animal Care and Use Committee of the Agency for Science, Technology, and Research (A*STAR) of Singapore. All mice were male, of the C57BL/6J genetic background and housed in a 12-hour light-dark cycle with access ad libitum to water and an NCD (Harlan 2018 Teklab Global 18% Protein Rodent Diet). For the DIO study, mice were fed an HFD with 60% of the calories from fat (D12492, Research Diets) from 8 wk to 17 wk of age, as indicated in the text.

### Ncls treatment

Ncls (Cat# N9789, Sigma-Aldrich) (1 mg/kg body weight) or veh was administered weekly by oral gavage consecutively for 7 wk to both NCD and HFD mice starting at 10 wk of age. Ncls treatment in HFD mice starts 2 wk later after the onset of HFD feeding to examine the effects of ncls on animals that are already slightly overweight. The veh solution consisted of 5% hydroxypropyl cyclodextrin (Sigma-Aldrich). The solutions were freshly prepared before each administration.

### Indirect calorimetry

Mice were placed in the Oxymax/Comprehensive Lab Animal Monitoring System (Columbus Instruments, Ohio, USA) consecutively for 3 d, including a 24-h acclimatization period, to determine VO_2_, VCO_2_, food intake, and spontaneous locomotor activity. The RER was calculated as the ratio of VCO_2_ / VO_2_. Energy expenditure was calculated according to the equation provided by Columbus Instruments at www.colinst.com. All values presented were normalized to the body weight or lean mass (for VO_2_). We used the mean value for each light and dark period to analyze statistical significance.

### Gene expression analysis

Total RNA was extracted from the indicated tissues using TRIzol reagent after the connective and surrounding tissues were removed. The RNA samples were then further purified using the RNeasy mini kit (Qiagen) and eluted in RNAsecure reagent (Ambion). Subsequently, RNA samples were quantified using a NanoDrop 1000 spectrophotometer (Thermo Scientific), and the RNA integrity was determined by an Agilent 2100 Bioanalyzer (Agilent Technologies). Quantitative PCR was performed (after reverse transcription of RNA) using Power SYBR Green PCR master mix (Applied Biosystems) with the 7900HT Fast Real-Time PCR System (Applied Biosystems). All gene expression data were normalized to *PPIA*. All primer sequences used in this study can be found in S4 Table. RNA sequencing (RNA-seq) was performed on the Illumina HiSeq 2000 platform at BGI (BGI Tech Solutions Co., China).

### RNA-Seq data analysis

All RNA-Seq datasets from the four major metabolic organs were aligned to the mouse genome (mm9) using the TopHat program (version 2.0.11). Alignments were performed as described [58]. The mapped reads were further analyzed using the Cufflinks program as previously described [59]. The expression levels of each transcripts were quantified as the FPKM based on the RefSeq database. To identify the DEGs across the four metabolic organs, we input the RNA-seq datasets (BAM files) into the Partek Genomic Suite program and set the following selection criteria in the program: Fold change > 2-fold, *p* value < 0.05, False Discovery Rate (FDR) < 0.05. In total, 1,532 DEGs were identified across the four metabolic organs and then a hierarchical clustering of these DEGs was performed according to their expression patterns. From the cluster enriched in skeletal muscle, we found in total 258 DEGs and listed these genes in S1 Table and used them for subsequent Gene Ontology (GO) analysis. GO analyses were performed with the online program DAVID [60] and the Partek Genomic Suite and Pathway programs.

### Cell culture

Mouse C2C12 myoblasts (American Type Culture Collection) were maintained in Dulbecco's modified Eagle’s medium (DMEM) supplemented with 20% fetal bovine serum and 0.5% chicken embryo extract. Human primary 36C15Q myoblasts were maintained in DMEM supplemented with 20% fetal bovine serum, 10% horse serum and 1% chicken embryo extract. To induce differentiation, myoblasts were incubated in DMEM containing 2% horse serum, and the induction medium was changed every 2 d.

### In vitro mitochondrial respiration measurement by Seahorse extracellular flux analyzer

Mitochondrial OCRs and ECARs were measured using the Seahorse extracellular flux analyzer XF24e (Seahorse Bioscience). C2C12 and 36C15Q cells were seeded at a density of 25,000 cells per cm^2^ in 24-well plates and differentiated as described in the main text. At day 3 of differentiation, 0.125 mM PA was added with or without 20 nM ncls for 48 h. On the day of the experiment, the cells were equilibrated for 1 h with 1x KHB buffer supplemented with 50 μM L-carnitine, 5 mM HEPES, and 2.5 mM D-glucose. OCR measurements were obtained before and after sequential additions of the ATPase inhibitor oligomycin (1.5 μM), the inner membrane uncoupler FCCP (1.5 μM), and the inhibitors of complex I and III—rotenone (1 μM) and antimycin A (1 μM). For measurement of FAO profiles, C2C12 and 36C15Q myotubes were incubated in substrate-limited medium overnight to prime the myotubes for utilization of exogenous fatty acids and assayed on the following day with the addition of 125 μM PA-BSA. Eto (40 uM) was used to inhibit FAO and to confirm the assay specificity. Oligomycin (1 μM), FCCP (1.6 μM), and Rotenone/Antimycin A (1 μM) were injected sequentially during the assay. ECAR glycolytic measurements were performed before and after sequential additions of oligomycin (1μM), FCCP (1 μM), and the glycolysis inhibitor 2-deoxy-D-glucose (100 mM). The OCR was normalized to the total protein in each well.

### Ex vivo mitochondrial respiration measurement in intact muscle fibers

The measurement of mitochondrial respiration in intact muscle fibers was performed according to the protocol entitled “Measuring mitochondrial respiration in intact skeletal muscle fibers” available from the Seahorse Bioscience website. Specifically, EDL muscle from all four groups of mice was carefully dissected to ensure that no mechanical damage was introduced to the muscle fibers. The muscle samples were then weighted and rinsed in prewarmed PBS. Each muscle was individually incubated in 2 ml of dissociation medium (Dulbecco’s Modified Eagle Medium (D-MEM) high glucose, no sodium pyruvate or phenol red (Invitrogen, #21063–029), gentamycin [50 μg/ml] (Sigma, #G1397), FBS [2%], and collagenase A [0.5 mg/ml] (Roche #11088785103) pH 7.2.) for 15 min at 37°C to slightly dissociate muscle fibers without separating them from each other (that would allow better plating in the well). Then, each individual muscle was plated on an XF24e islet capture microplate (Seahorse Bioscience) using a dissecting microscope to ensure no damage was introduced to the muscle fibers during the transfer. Islet capture screen was then placed on top of the muscle in the well. Then, the rate of FAO was determined according to the protocol entitled “Conducting a Fatty Acid Oxidation Assay on the XF Analyzer” also available from the Seahorse Bioscience website. In the assay, EDL muscle fibers from all four groups of mice were rinsed twice with assay medium without collagenase (1x KHB assay medium: NaCl, 111 mM; KCl, 4.7 mM; MgSO4, 2 mM; Na2HPO4, 1.2 mM; Glucose, 2.5 mM; Carnitine 0.5 mM), then the OCRs were measured after the addition of PA-BSA (125 μM). Assay was performed with 500 μl of assay medium per well for 35 min. The value of the OCR was normalized to the mass of EDL muscle in mg.

### cAMP concentration

cAMP quantification was performed at 48 h post ncls treatment using the cAMP Complete ELISA Kit (#ADI-900-163, Enzo) as indicated by manufacturer’s protocol with minor modifications. Briefly, ncls- or veh-treated C2C12 myotubes were lysed in 0.1 M HCl containing 0.1% Triton X-100 for 5 min at room temperature, centrifuged at 1,000 g for 3 min to pellet the cellular debris, and then 100 μl of cell lysate (supernatant) was used for the subsequent ELISA assay. cAMP concentration was determined by ELISA assay through measuring optical densities at 405 nm using a spectrometry plate reader (Tecan), followed by calculations based on cAMP standard utilizing a four-parameter logistic curve fitting program (GraphPad Software, Inc., San Diego, CA, USA). Data were normalized to the amount of protein present in the supernatant as measured by the BCA assay.

### ADP/ATP ratio

ADP/ATP ratio was measured at 48 h post ncls treatment using the ADP/ATP Ratio Assay Kit (#ab65313, Abcam) as per the manufacturer’s instructions with minor changes. Briefly, ncls- or veh-treated C2C12 myotubes were lysed in nucleotide-releasing buffer for 5 min at room temperature, centrifuged at 10,000 g for 1 min to pellet insoluble materials, and then the supernatant was used for subsequent measurement. ATP or ADP levels in C2C12 myotubes were determined by measuring luminescence in the absence or presence of ADP converting enzyme using a luminometer (Tecan). ADP/ATP ratio was calculated and normalized to the amount of protein present in the supernatant as measured by the BCA assay.

### MMP

The membrane-permeant JC-1 dye (Molecular probe) was used to monitor MMP in ncls- or veh-treated C2C12 myotubes at 72 h post ncls treatment. In brief, myotubes after various treatments were stained with 1 μM of JC-1 for 30 min at 37°C and then examined using fluorescence microscopy at Ex 540 nm / Em 570 nm and Ex 485 nm / Em 535 nm. Quantitative changes of MMP after ncls treatment were determined by measuring fluorescence intensities of JC-1 aggregates (Ex 535 nm / Em 595 nm) and JC-1 monomer (Ex 485 nm / Em 535 nm). For flow cytometric analysis, C2C12 myotubes (10^6^ cells / ml) were stained with 1 μM of JC-1 for 30 min at 37°C, trypsinized, and washed with PBS before JC-1 signals were analyzed on a flow cytometer (Beckman Coulter) using 488 nm excitation with 530 nm and 585 nm bandpass emission filters.

### Hydrogen peroxide concentration

H2O2 concentration was determined at 48 h post ncls treatment using the Amplex Red Hydrogen Peroxide/Peroxidase Assay Kit (#A22188, Invitrogen) in ncls- or veh-treated C2C12 myotubes. Briefly, C2C12 myotubes (10^6^ cells / ml) were incubated with 50 μM Amplex Red reagent plus 0.1 U / ml horseradish peroxidase for 30 min in darkness, and then the concentration of H2O2 was measured as per manufacturer’s instructions on a fluorescence plate reader (Tecan).

### Statistical analysis

Results are expressed as the mean values ± SEM or the averages ± standard deviation. The datasets were analyzed for statistical significance between groups using either two-tailed Student’s *t*-test or 2-way ANOVA. Methods of statistical analysis are provided in S1 Data. Statistical analyses (except RNA-seq) were performed using GraphPad software, version 5 or Excel. A *p*-value < 0.05 was considered significant.

### Accession numbers

RNA-seq datasets were deposited into the Gene Expression Omnibus under accession number GSE63268.

## Supporting information

S1 Fig Effects of ncls treatment on the fat mass and lean mass in HFD and NCD mice. (A) Percentages of fat mass in total body weight of the ncls- or veh-treated mice on either an HFD or an NCD over a 9-wk period. (B) Percentages of fat mass and lean mass in total body weight of the mice after 6 wk of ncls or veh treatment (Week 8). * *p* < 0.05, ** *p* < 0.01, *** *p* < 0.001. Underlying data and method of statistical analysis are provided in S1 Data.
(TIF)

S2 Fig Ncls promotes fatty acid metabolism in HFD mice. (A) Carbon dioxide production (VCO_2_) of HFD and NCD mice after 6 wk of ncls or veh administration. (B) RER of the mice described in (A). Ncls treatment significantly reduced the RER in HFD mice, indicating increased fatty acid metabolism in HFD-ncls mice. **** *p* < 0.0001. Underlying data and method of statistical analysis are provided in S1 Data.
(TIF)

S3 Fig Ncls does not affect food intake and fecal energy output in mice. (A) Cumulative food intake of HFD and NCD mice over a 48-h period after 6 wk of ncls or veh administration. (B) Average daily food intake of the mice described in (A). (C) Absolute daily caloric intake of the mice described in (A). (D) Feces production, (E) fecal energy content as determined by bomb calorimeter, and (F) total fecal energy output per day of the mice described in (A). **** *p* < 0.0001. Underlying data and method of statistical analysis are provided in S1 Data.
(TIF)

S4 Fig Effects of ncls treatment on mouse behavior. Mouse anxiety was evaluated by behavior tests including the (A) limb clasping test, (B) open field test, and (C) elevated plus maze test. Representative mouse trajectories for HFD-veh and HFD-ncls mice in the open field test were shown in panel (B). Values represent means ± SEM. Underlying data and method of statistical analysis are provided in S1 Data.
(TIF)

S5 Fig Correlation of skeletal muscle RNA-seq datasets between NCD-veh and NCD-ncls mice. The transcriptomic profiles of skeletal muscle of NCD-veh and NCD-ncls mice showed very high positive correlation (Pearson correlation coefficients, *R* = 0.999), suggesting ncls treatment did not lead to major transcriptional changes in NCD mice at the genomic level. Underlying data and method of statistical analysis are provided in S1 Data.
(TIF)

S6 Fig Effects of ncls on muscle strength and muscle mass. (A) Grip strength performance of mice at the beginning of the study (Baseline) and after 7 wk of ncls or veh treatment (Week 7). (B-F) Mass of (B) EDL, (C) soleus, (D) TA, (E) Gastr., and (F) quadricep muscles from mice treated for 7 wk with ncls or veh on either an HFD or an NCD. * *p* < 0.05, ** *p* < 0.01, *** *p* < 0.001, **** *p* < 0.0001. Underlying data and method of statistical analysis are provided in S1 Data.
(TIF)

S7 Fig Effects of ncls on muscle fiber area and fiber type in quadriceps of NCD and HFD mice. (A) Quadricep muscles were harvested from mice treated with ncls or veh on either an HFD or an NCD. Muscle sections were H&E stained, and the CSA of muscle fibers were quantified using ImageJ. (B) Muscle fiber type transition in the quadriceps upon ncls treatment in both HFD and NCD mice. Serial cryosections of quadricep muscles were immunostained with specific MHC antibodies and the percentages of each fiber type (MHC I, MHC IIa, and MHC IIb) were determined. * *P* < 0.05. (C) Representative images for MHC immunostaining of quadriceps muscle from mice treated with ncls or veh on either an HFD or an NCD. Scale bar, 100 μm. Underlying data and method of statistical analysis are provided in S1 Data.
(TIF)

S8 Fig Ncls promotes the expression of oxidative metabolism genes but represses glycolytic and fatty acid synthesis genes in quadriceps muscle of HFD mice. (A) The top 3 highly enriched categories of DEGs (>1.4-fold change) in quadricep muscles from HFD-ncls, NCD-veh and HFD-veh mice. (B) Expression patterns of representative genes from the “metabolic process” category, as described in (A). Underlying data and method of statistical analysis are provided in S1 Data.
(TIF)

S9 Fig Effects of ncls on metabolic gene expression in WAT, BAT, liver, and heart. Relative mRNA expression of metabolic genes in (A) WAT, (B) BAT, (C), liver and (D) heart from mice treated with ncls or veh on either an HFD or an NCD. Underlying data and method of statistical analysis are provided in S1 Data.
(TIF)

S10 Fig Ncls slightly enhances mitochondrial respiration in both murine myotubes and primary human myotubes in the absence of PA treatment. Relative mRNA expression of selected metabolic genes in (A) murine myotubes (C2C12) and (B) primary human myotubes (36C15Q) after treatment with or without 20 nM ncls for 48 h. The values of the untreated myotubes were arbitrarily set as one. Mitochondrial respiration was evaluated with a Seahorse extracellular flux analyzer. The OCR output for the BR, PL, AP, MR, and SRC of ncls-treated (C) C2C12 and (D) 36C15Q myotubes were shown as bar graphs. The OCR was normalized to the total protein per well. OCR profiles of (E) C2C12 and (F) 36C15Q myotubes with or without 20 nM ncls treatment were determined by a Seahorse extracellular flux analyzer. Vertical dashed lines indicate the time points of oligomycin (Oligo, 1 μM), FCCP (1.6 μM) and Rotenone/Antimycin A (Ret/A, 1 μM) injection. * *p* < 0.05, *** *p* < 0.001. Underlying data and method of statistical analysis are provided in S1 Data.
(TIF)

S11 Fig Ncls partially activates AMPK signaling in C2C12 myotubes without PA treatment but exerts no detectable effects on AMPK signaling in the skeletal muscle of NCD mice. (A) Ncls treatment led to slight increases in pAMPKα and pACC2 in C2C12 myotubes without PA treatment. Western blots of total AMPKα and ACC2 were used for loading controls and subsequent densitometry measurement. Ratios of phospho- to total- AMPKα and ACC2 were shown as bar graphs below the representative immuno-blotting images of two independent experiments performed in duplicates. (B) Ncls treatment had no detectable effects on pAMPKα and pACC2 as evaluated by western blotting in quadricep muscles from NCD mice. Protein levels of AMPKα and ACC2 were evaluated by western blotting in parallel. glyceraldehyde 3-phosphate dehydrogenase (GAPDH) was included as a loading control. Underlying data and method of statistical analysis are provided in S1 Data.
(TIF)

S1 Table List of the 258 DEGs in the skeletal muscle of HFD-veh, NCD-veh, and HFD-ncls mice (>2-fold change). (XLSX)

S2 Table Gene list of the enriched “Muscle protein” category in the up-regulated genes in the skeletal muscle of HFD-ncls mice (>2-fold change). (DOCX)

S3 Table Expression levels of the genes described in S2 Table from the RNA-seq analysis. (DOCX)

S4 Table Primers for qRT-PCR. (DOCX)

S1 Text Supporting materials and methods. (DOCX)

S1 Data Contains underlying data for Figs 1A–1C,1E, 1H; 2B–2I; 3B, 3D–3F, 3H; 4D–4E; 5A, 5C–5I, 5K; 6A, 6B, 6E–6J; 7A, 7B, 7G and 8A–8D; S1A, S1B; S2A,S2B; S3A–S3F; S4B,S4C; S5; S6A–S6F; S7A,S7B; S8A; S9A–S9D; S10A–S10F and S11A Figs. (XLSX)

## Abbreviations

*Acadl*: acyl-CoA dehydrogenase, long chain
ACC: acetyl-CoA carboxylase
*Acsl1*: acyl-CoA synthetase long-chain family member 1
ADP: adenosine diphosphate
AICAR: 5-Aminoimidazole-4-carboxamide ribonucleotide
AMPK: AMP-activated protein kinase
AP: ATP production
AUC: area under curve
BAT: brown adipose tissue
BR: basal rate
CaMKK: Ca(2+)/calmodulin-dependent protein kinase kinase
*Cpt1b*: carnitine palmitoyltransferase 1B
*Cs*: citrate synthase
CSA: cross-sectional area
DEG: differentially expressed genes
DIO: diet-induced obesity
ECAR: extracellular acidification rate
EDL: extensor digitorum longus
Eto: etomoxir
FAO: fatty acid oxidation
*Fasn*: fatty acid synthase
*Fatp1*: fatty acid transport protein 1
FCCP: carbonilcyanide p-triflouromethoxyphenylhydrazone
FFA: free fatty acid
FPKM: fragments per kilobase of exon per million fragments mapped
GAPDH: glyceraldehyde 3-phosphate dehydrogenase
GSIS: glucose-stimulated insulin secretion
GTT: glucose tolerance test
H&E: hematoxylin & eosin
HFD: high-fat diet
ITT: insulin tolerance test
LKB1: liver kinase B1
MHC: myosin heavy chain
MMP: mitochondrial membrane potential
MR: maximal respiration
MRI: magnetic resonance imaging
NCD: normal chow diet
ncls: narciclasine
NMR: nuclear magnetic resonance
OCR: oxygen consumption rates
PA: palmitate
pACC2: phospho-ACC2
pAMPKα: phospho-AMPKα
*Pfkl*: phosphofructokinase
PL: proton leak
*Ppara*: peroxisome proliferator-activated receptor alpha
qRT-PCR: quantitative reverse transcription polymerase chain reaction
RER: respiratory exchange ratio
ROS: reactive oxygen species
*Scd1*: stearoyl-CoA desaturase
SEM: standard error of the mean
SRC: spare respiratory capacity
TA: tibialis anterior
*Tfam*: mitochondrial transcription factor A
TG: triglyceride
veh: vehicle
WAT: white adipose tissue
