# Supplementary figures and images for: Narciclasine attenuates diet-induced obesity by promoting oxidative metabolism in skeletal muscle
Source: PLoS Biol. 2017 Feb 16;15(2):e1002597. doi: 10.1371/journal.pbio.1002597 (PMC5331945; doi:10.1371/journal.pbio.1002597)

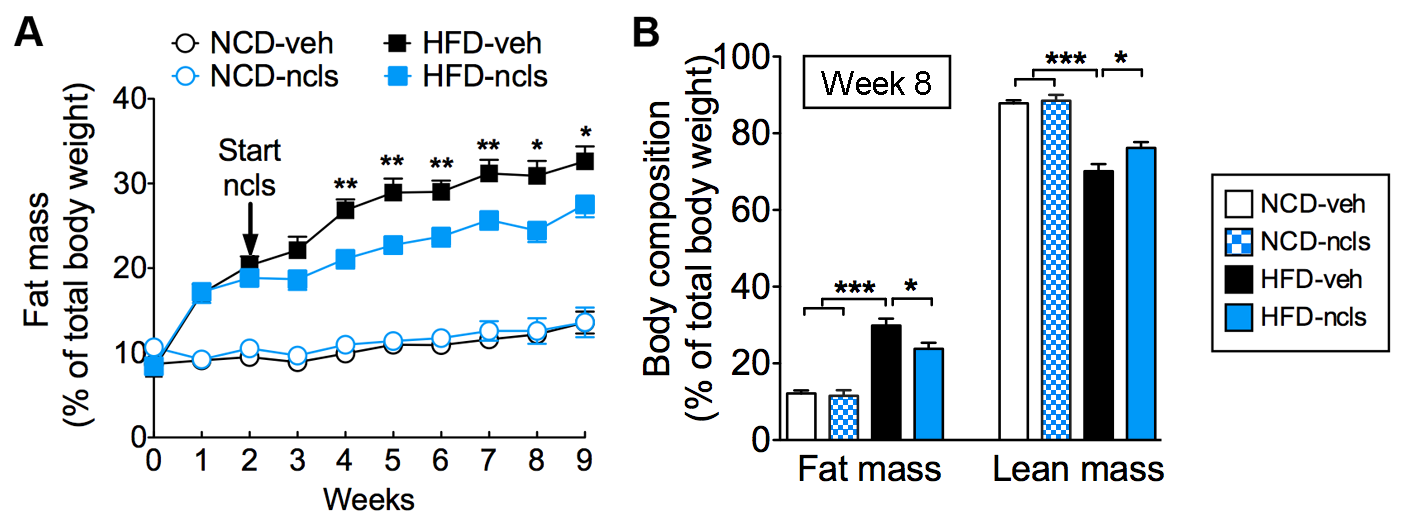

Supplement: S1 Fig — (A) Percentages of fat mass in total body weight of the ncls- or veh-treated mice on either an HFD or an NCD over a 9-wk period. (B) Percentages of fat mass and lean mass in total body weight of the mice after 6 wk of ncls or veh treatment (Week 8). * p < 0.05, ** p < 0.01, *** p < 0.001. Underlying data and method of statistical analysis are provided in S1 Data. (TIF) [file pbio.1002597.s001.tif]

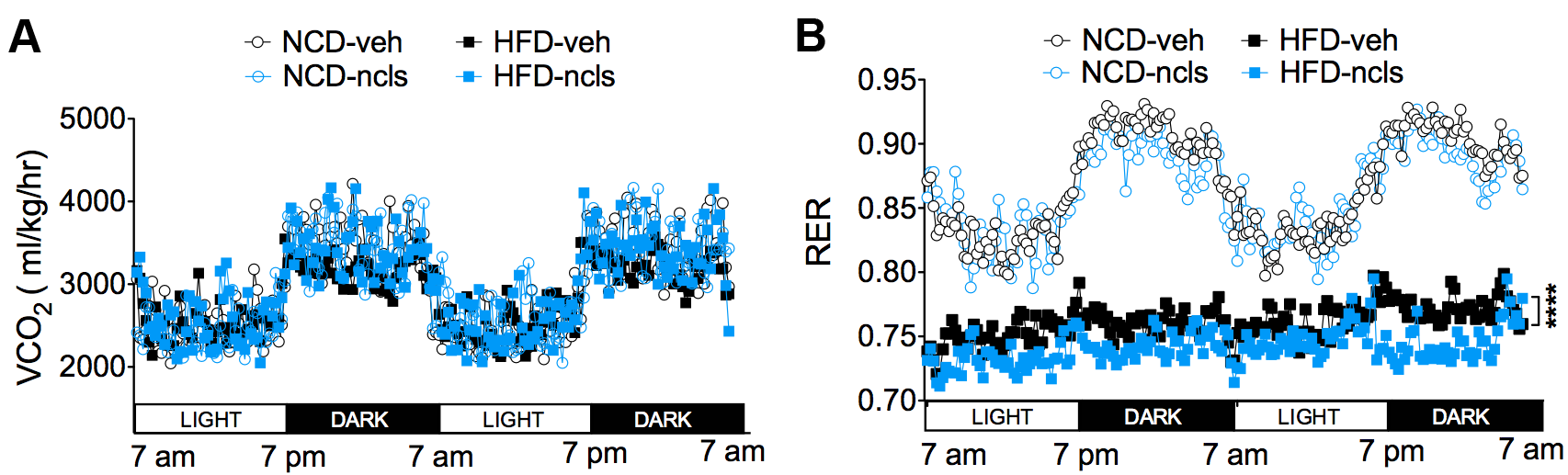

Supplement: S2 Fig — (A) Carbon dioxide production (VCO2) of HFD and NCD mice after 6 wk of ncls or veh administration. (B) RER of the mice described in (A). Ncls treatment significantly reduced the RER in HFD mice, indicating increased fatty acid metabolism in HFD-ncls mice. **** p < 0.0001. Underlying data and method of statistical analysis are provided in S1 Data. (TIF) [file pbio.1002597.s002.tif]

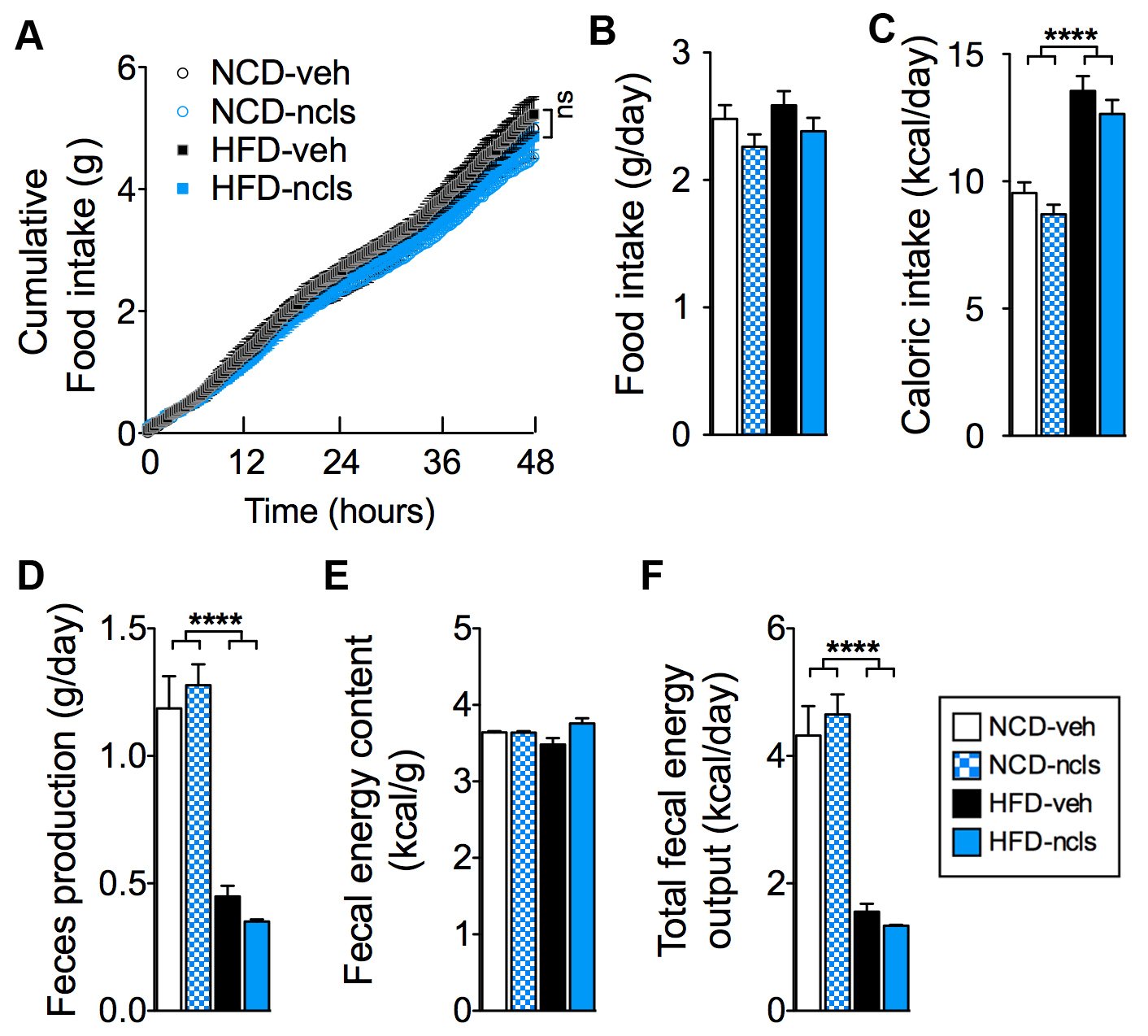

Supplement: S3 Fig — (A) Cumulative food intake of HFD and NCD mice over a 48-h period after 6 wk of ncls or veh administration. (B) Average daily food intake of the mice described in (A). (C) Absolute daily caloric intake of the mice described in (A). (D) Feces production, (E) fecal energy content as determined by bomb calorimeter, and (F) total fecal energy output per day of the mice described in (A). **** p < 0.0001. Underlying data and method of statistical analysis are provided in S1 Data. (TIF) [file pbio.1002597.s003.tif]

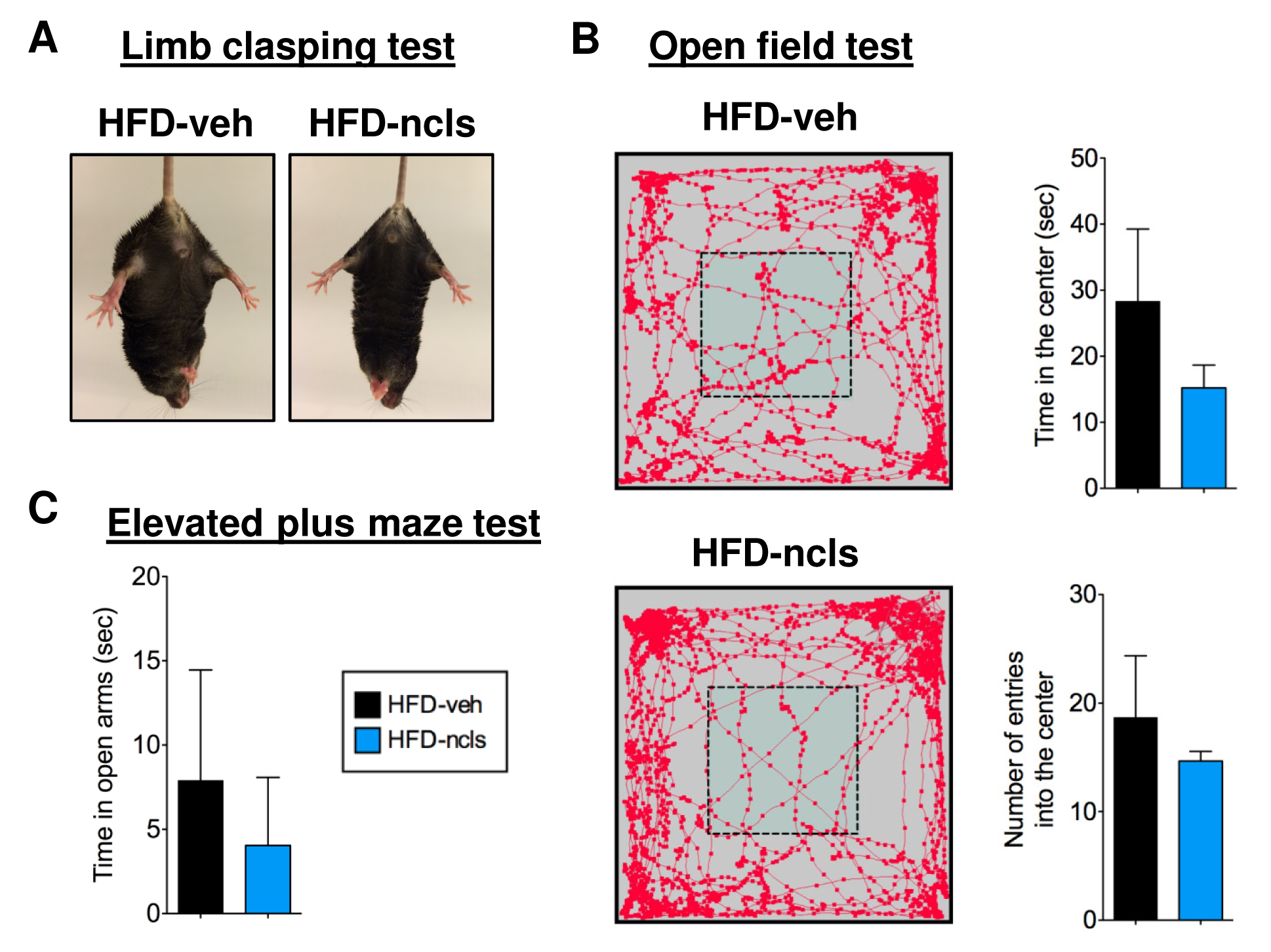

Supplement: S4 Fig — Mouse anxiety was evaluated by behavior tests including the (A) limb clasping test, (B) open field test, and (C) elevated plus maze test. Representative mouse trajectories for HFD-veh and HFD-ncls mice in the open field test were shown in panel (B). Values represent means ± SEM. Underlying data and method of statistical analysis are provided in S1 Data. (TIF) [file pbio.1002597.s004.tif]

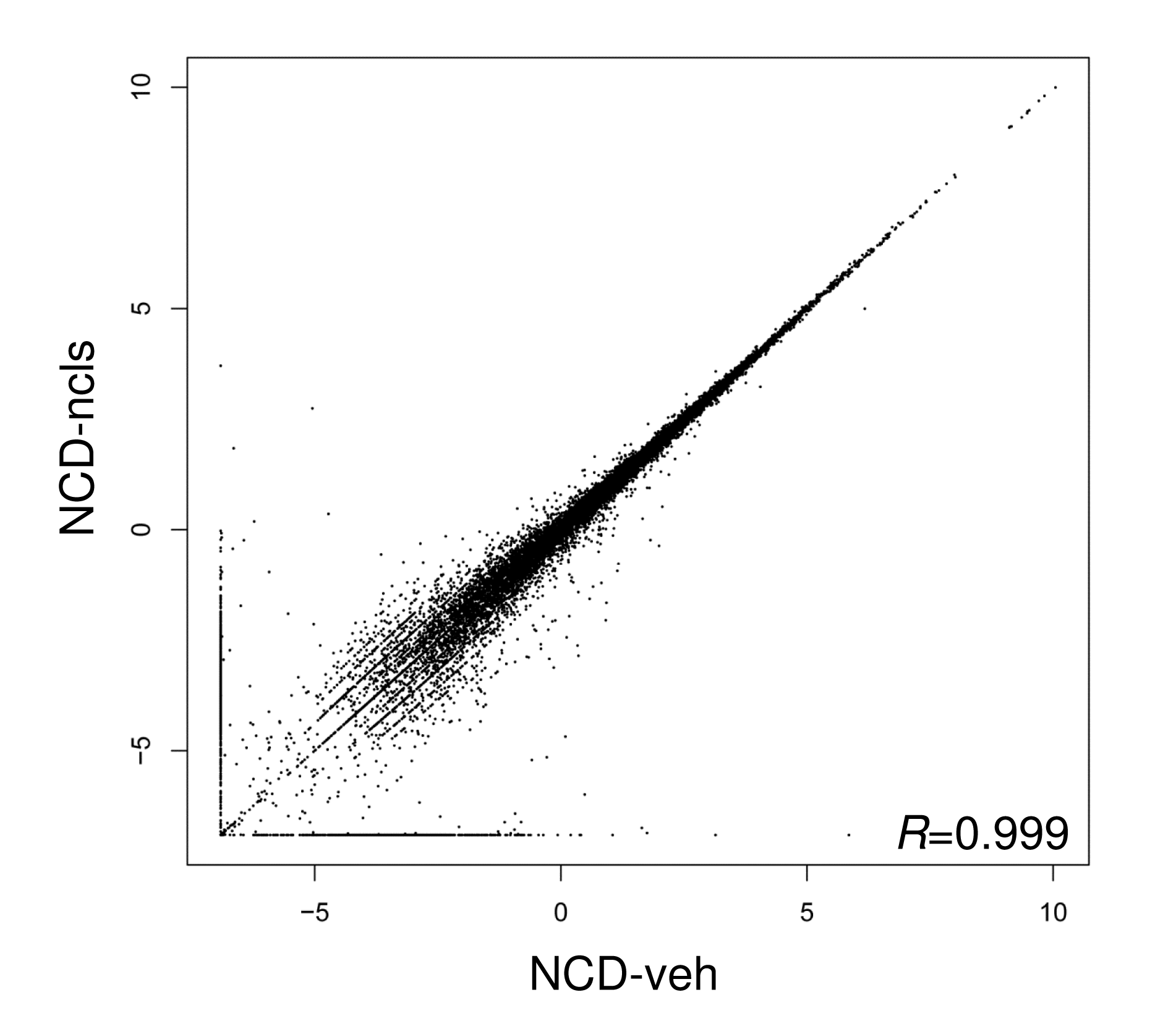

Supplement: S5 Fig — The transcriptomic profiles of skeletal muscle of NCD-veh and NCD-ncls mice showed very high positive correlation (Pearson correlation coefficients, R = 0.999), suggesting ncls treatment did not lead to major transcriptional changes in NCD mice at the genomic level. Underlying data and method of statistical analysis are provided in S1 Data. (TIF) [file pbio.1002597.s005.tif]

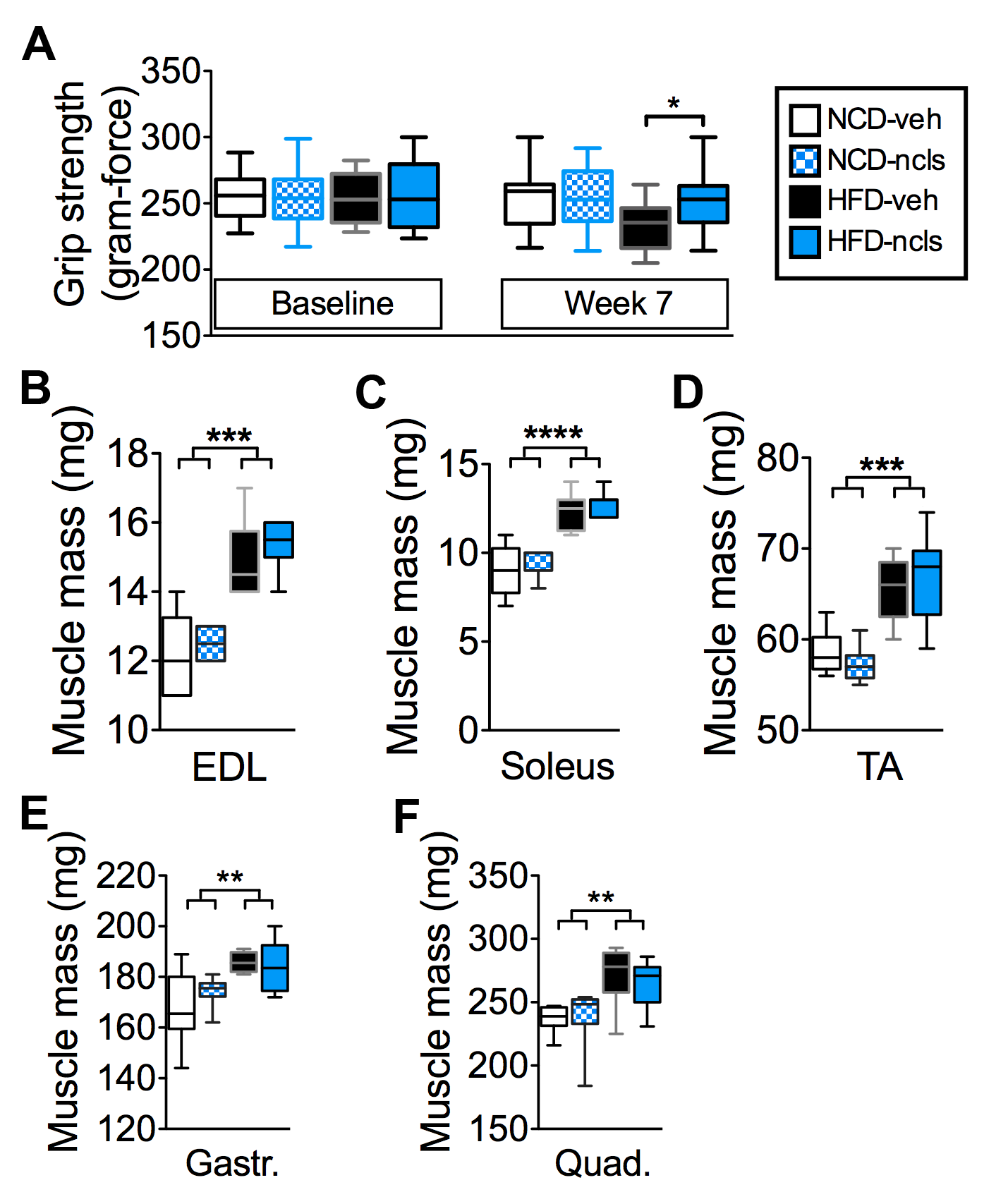

Supplement: S6 Fig — (A) Grip strength performance of mice at the beginning of the study (Baseline) and after 7 wk of ncls or veh treatment (Week 7). (B-F) Mass of (B) EDL, (C) soleus, (D) TA, (E) Gastr., and (F) quadricep muscles from mice treated for 7 wk with ncls or veh on either an HFD or an NCD. * p < 0.05, ** p < 0.01, *** p < 0.001, **** p < 0.0001. Underlying data and method of statistical analysis are provided in S1 Data. (TIF) [file pbio.1002597.s006.tif]

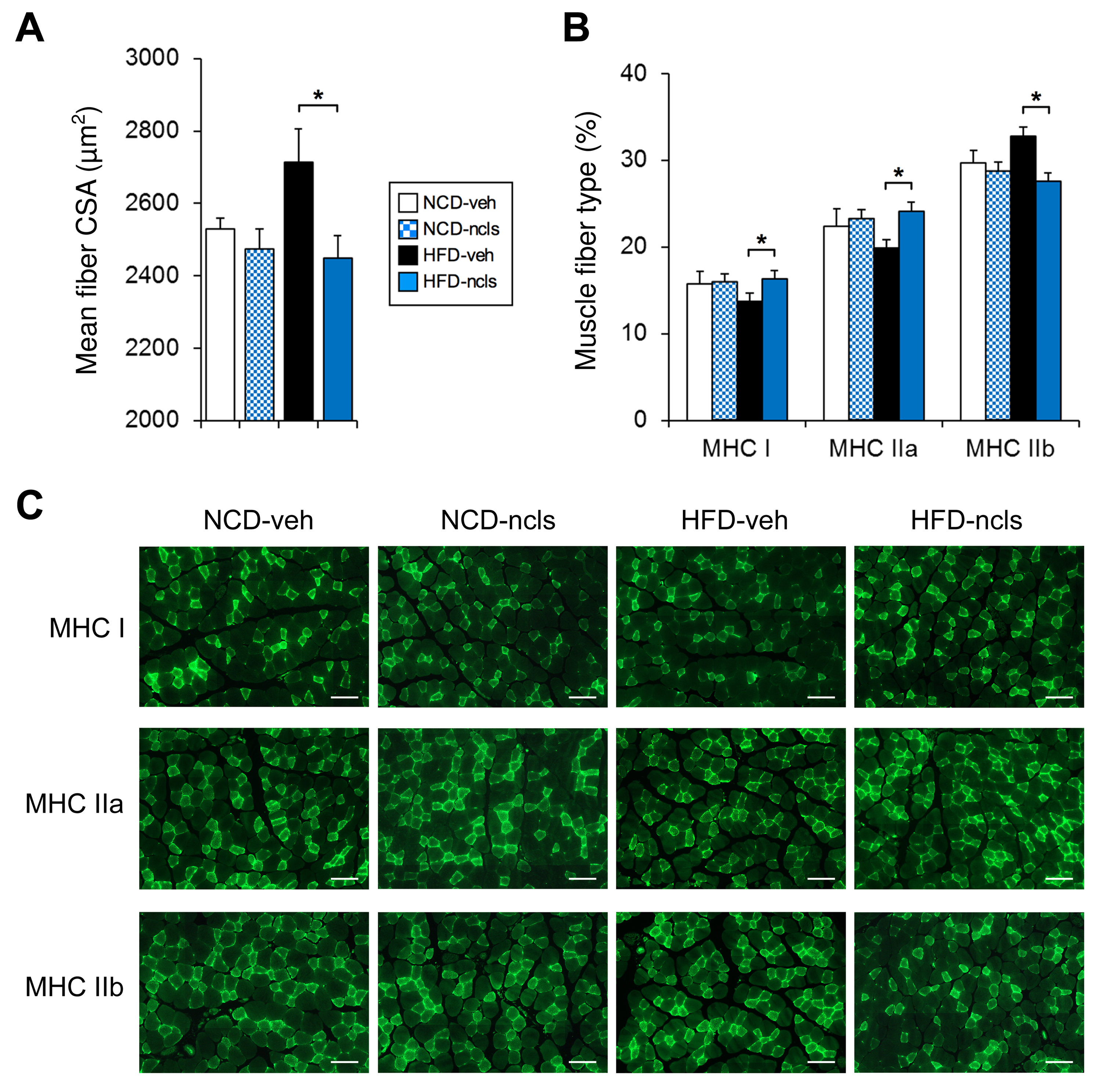

Supplement: S7 Fig — (A) Quadricep muscles were harvested from mice treated with ncls or veh on either an HFD or an NCD. Muscle sections were H&E stained, and the CSA of muscle fibers were quantified using ImageJ. (B) Muscle fiber type transition in the quadriceps upon ncls treatment in both HFD and NCD mice. Serial cryosections of quadricep muscles were immunostained with specific MHC antibodies and the percentages of each fiber type (MHC I, MHC IIa, and MHC IIb) were determined. * P < 0.05. (C) Representative images for MHC immunostaining of quadriceps muscle from mice treated with ncls or veh on either an HFD or an NCD. Scale bar, 100 μm. Underlying data and method of statistical analysis are provided in S1 Data. (TIF) [file pbio.1002597.s007.tif]

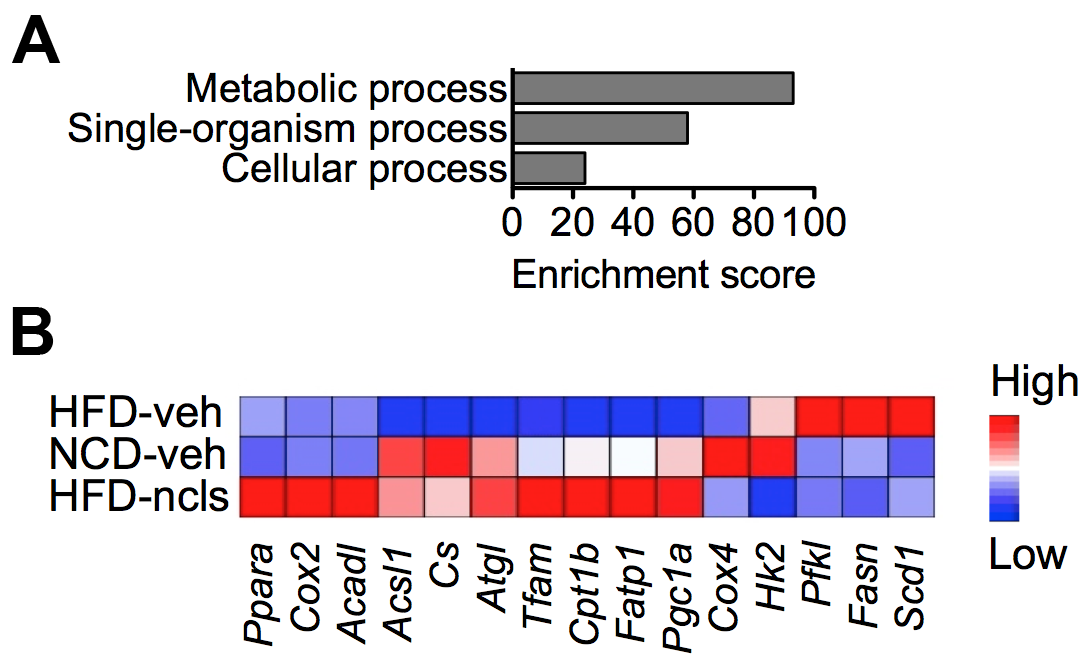

Supplement: S8 Fig — (A) The top 3 highly enriched categories of DEGs (>1.4-fold change) in quadricep muscles from HFD-ncls, NCD-veh and HFD-veh mice. (B) Expression patterns of representative genes from the “metabolic process” category, as described in (A). Underlying data and method of statistical analysis are provided in S1 Data. (TIF) [file pbio.1002597.s008.tif]

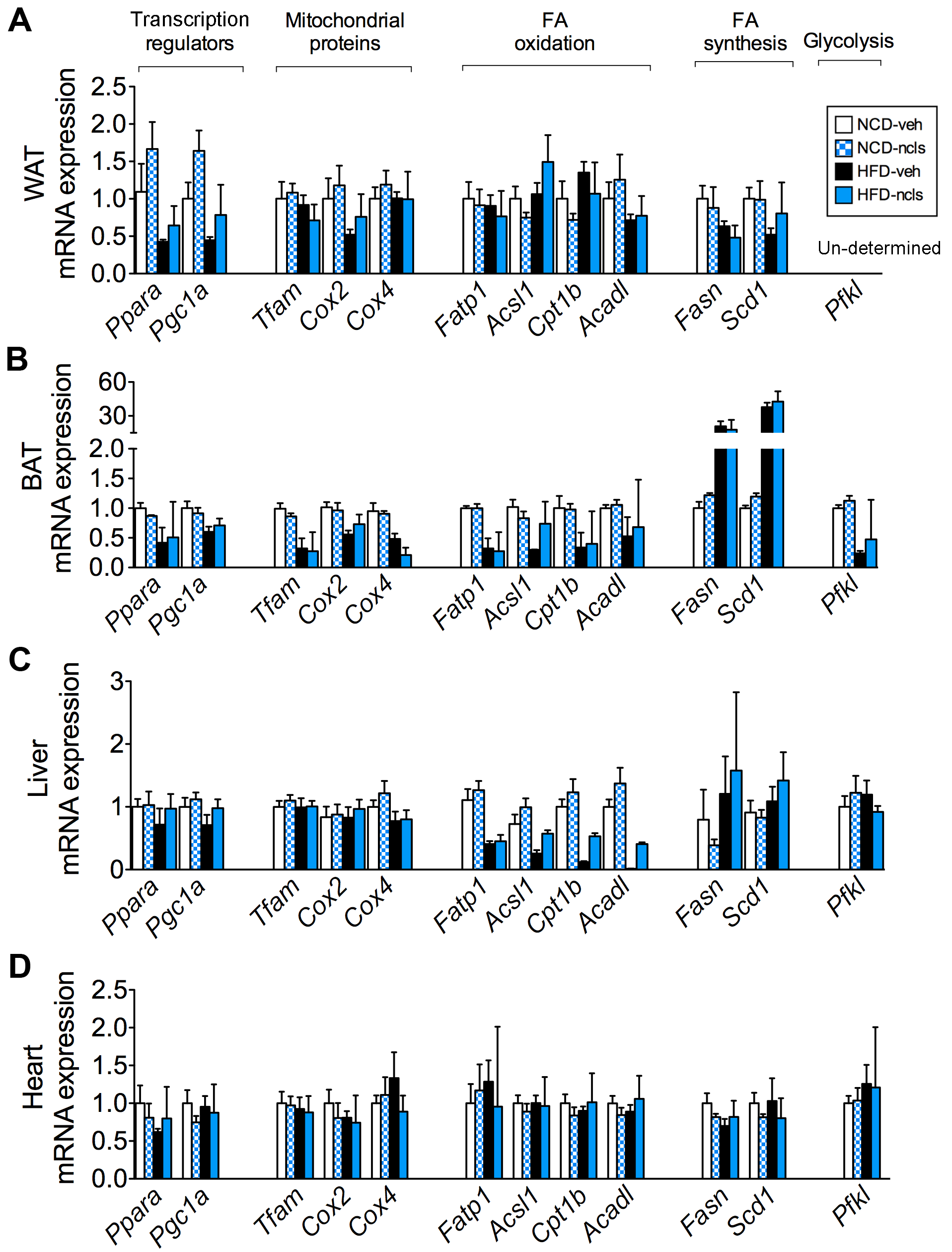

Supplement: S9 Fig — Relative mRNA expression of metabolic genes in (A) WAT, (B) BAT, (C), liver and (D) heart from mice treated with ncls or veh on either an HFD or an NCD. Underlying data and method of statistical analysis are provided in S1 Data. (TIF) [file pbio.1002597.s009.tif]

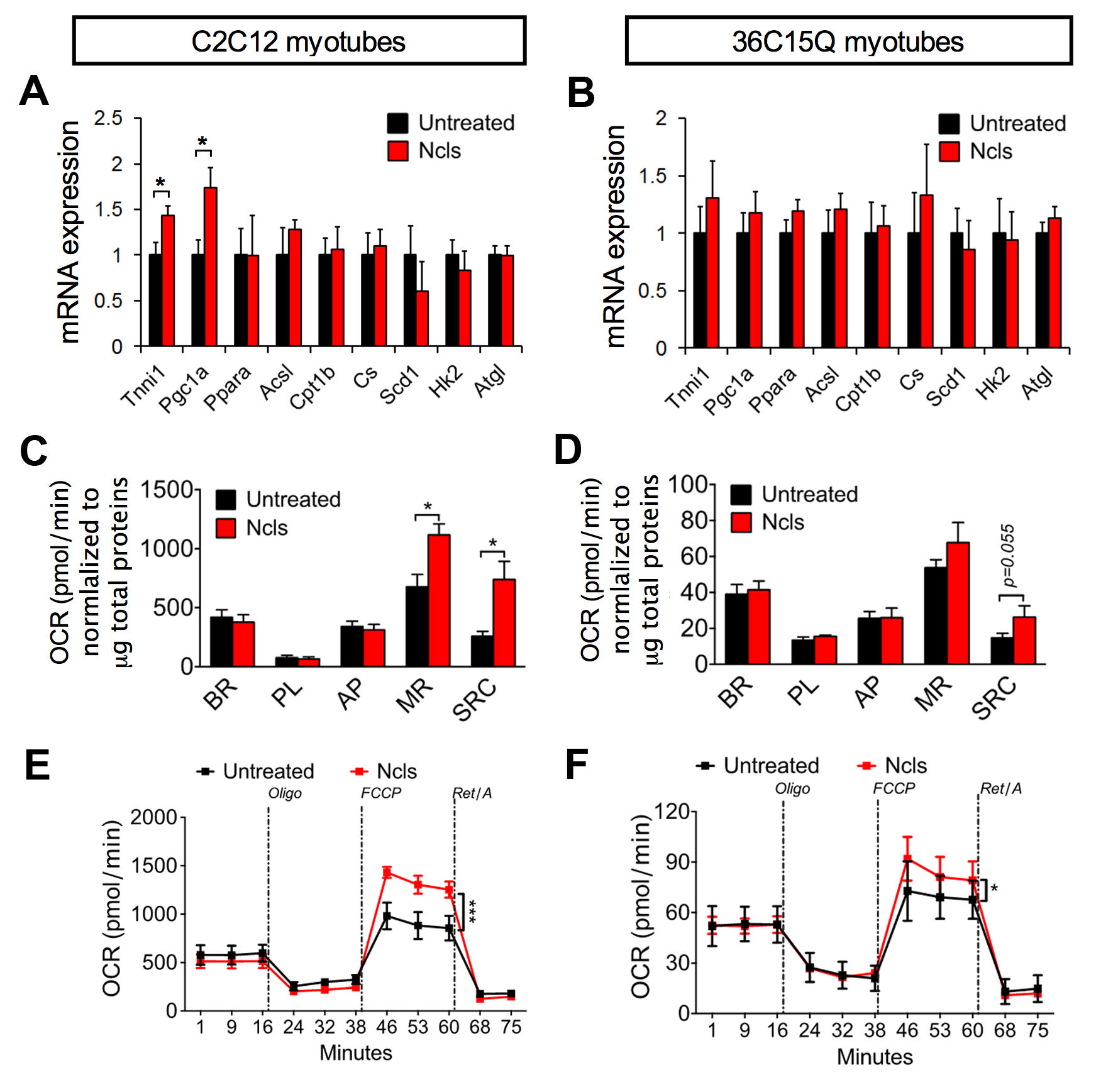

Supplement: S10 Fig — Relative mRNA expression of selected metabolic genes in (A) murine myotubes (C2C12) and (B) primary human myotubes (36C15Q) after treatment with or without 20 nM ncls for 48 h. The values of the untreated myotubes were arbitrarily set as one. Mitochondrial respiration was evaluated with a Seahorse extracellular flux analyzer. The OCR output for the BR, PL, AP, MR, and SRC of ncls-treated (C) C2C12 and (D) 36C15Q myotubes were shown as bar graphs. The OCR was normalized to the total protein per well. OCR profiles of (E) C2C12 and (F) 36C15Q myotubes with or without 20 nM ncls treatment were determined by a Seahorse extracellular flux analyzer. Vertical dashed lines indicate the time points of oligomycin (Oligo, 1 μM), FCCP (1.6 μM) and Rotenone/Antimycin A (Ret/A, 1 μM) injection. * p < 0.05, *** p < 0.001. Underlying data and method of statistical analysis are provided in S1 Data. (TIF) [file pbio.1002597.s010.tif]

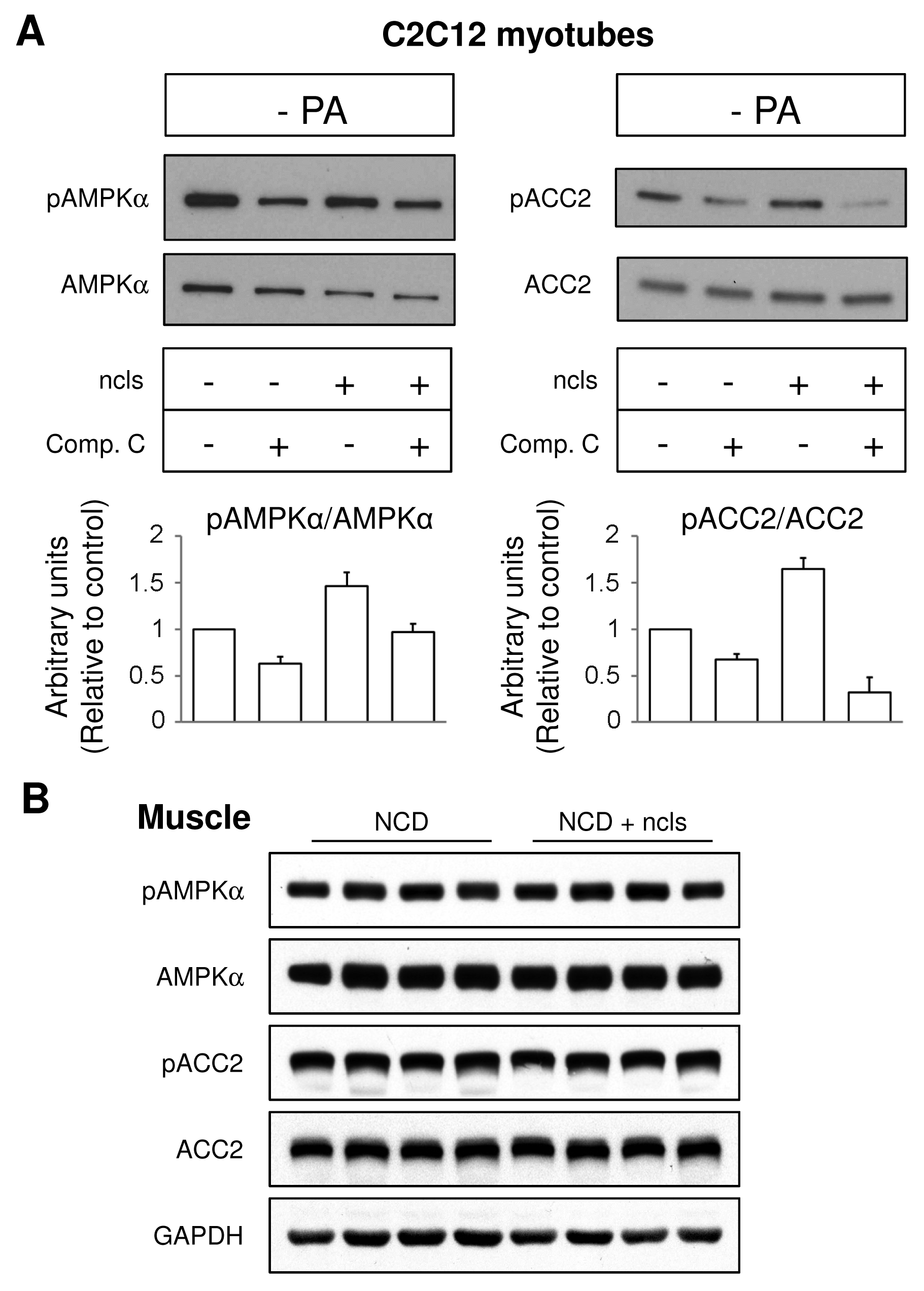

Supplement: S11 Fig — (A) Ncls treatment led to slight increases in pAMPKα and pACC2 in C2C12 myotubes without PA treatment. Western blots of total AMPKα and ACC2 were used for loading controls and subsequent densitometry measurement. Ratios of phospho- to total- AMPKα and ACC2 were shown as bar graphs below the representative immuno-blotting images of two independent experiments performed in duplicates. (B) Ncls treatment had no detectable effects on pAMPKα and pACC2 as evaluated by western blotting in quadricep muscles from NCD mice. Protein levels of AMPKα and ACC2 were evaluated by western blotting in parallel. glyceraldehyde 3-phosphate dehydrogenase (GAPDH) was included as a loading control. Underlying data and method of statistical analysis are provided in S1 Data. (TIF) [file pbio.1002597.s011.tif]
