# Supplementary material for: Narciclasine attenuates diet-induced obesity by promoting oxidative metabolism in skeletal muscle
Source: PLoS Biol. 2017 Feb 16;15(2):e1002597. doi: 10.1371/journal.pbio.1002597 (PMC5331945; doi:10.1371/journal.pbio.1002597)
Supplement: S2 Table — (DOCX) [file pbio.1002597.s013.docx]

**S2 Table. Gene list of the enriched “Muscle protein” category in the up-regulated genes in skeletal muscle of HFD-ncls mice (> 2-fold change).**

| **Category** | **Term** | ***P-*Value** | **Genes** | **Counts** | **List Total** | **Fold Enrichment** | **FDR** |
| --- | --- | --- | --- | --- | --- | --- | --- |
| SP_PIR_ KEYWORDS | Muscle protein | 2.55E-07 | *Tnnt1*  *Tnni1*  *Myl2*  *Tnnc1*  *Myl3*  *Actc1*  *Myh7* | 7 | 96 | 26.04 | 3.06E-04 |
