# Supplementary material for: Narciclasine attenuates diet-induced obesity by promoting oxidative metabolism in skeletal muscle
Source: PLoS Biol. 2017 Feb 16;15(2):e1002597. doi: 10.1371/journal.pbio.1002597 (PMC5331945; doi:10.1371/journal.pbio.1002597)
Supplement: S3 Table — (DOCX) [file pbio.1002597.s014.docx]

**S3 Table. Expression levels of the genes described in S2 Table from the RNA-seq analysis.**

| **Gene Name** | **Gene Expression Levels (FPKM)** | | | **Fold Changes** | |
| --- | --- | --- | --- | --- | --- |
|  | **HFD-veh** | **HFD-ncls** | **NCD-veh** | **HFD-ncls/ HFD-veh** | **NCD-veh/ HFD-veh** |
| *Tnnt1* | 0.001 | 3.29012 | 2.6882 | 3290.12 | 2688.2 |
| *Tnni1* | 0.0839811 | 2.17908 | 1.41765 | 25.94726671 | 16.88058385 |
| *Myl2* | 0.734632 | 16.174 | 10.5421 | 22.01646539 | 14.35017805 |
| *Tnnc1* | 0.210536 | 4.56394 | 4.64237 | 21.67771782 | 22.05024319 |
| *Myl3* | 0.807421 | 9.58909 | 11.6573 | 11.87619594 | 14.43769731 |
| *Actc1* | 146.183 | 407.324 | 642.004 | 2.786397871 | 4.391782902 |
| *Myh7* | 5.10704 | 11.677 | 13.4284 | 2.286451643 | 2.629390018 |
