# Supplementary material for: Narciclasine attenuates diet-induced obesity by promoting oxidative metabolism in skeletal muscle
Source: PLoS Biol. 2017 Feb 16;15(2):e1002597. doi: 10.1371/journal.pbio.1002597 (PMC5331945; doi:10.1371/journal.pbio.1002597)
Supplement: S4 Table — (DOCX) [file pbio.1002597.s015.docx]

**S4 Table. Primers for qRT-PCR.**

| Primer | Sequences (5’ - 3’) | References |
| --- | --- | --- |
| Myl2-F | ATCGACAAGAATGACCTAAGGGA | [[1](#_ENREF_1)] |
| Myl2-R | ATTTTTCACGTTCACTCGTCCT |  |
| Myl3-F | TGGGGAAGCCAAAACAGGAAG | [[2](#_ENREF_2)] |
| Myl3-R | AGCCATCAGTTTCTCTACCTCA |  |
| Myh7-F | GTTTCCTTACTTGCTACCCTCAG | [[3](#_ENREF_3)] |
| Myh7-R | TGGATTCTCAAACGTGTCTAGTG |  |
| Tnni1-F | TGAAGCCAAATGCCTCCACAACAC | [[4](#_ENREF_4)] |
| Tnni1-R | ACACCTTGTGCTTAGAGCCCAGTA |  |
| Tnnc1-F | AGCTCATGAAGGACGGTGACAAGA | [[4](#_ENREF_4)] |
| Tnnc1-R | AACCGTGCAAGACCAGCATCTACT |  |
| Tnnt1-F | TGGATCCACCAGCTGGAATCAGAA | [[4](#_ENREF_4)] |
| Tnnt1-R | GCTGATGCGGTTGTAGAGCACATT |  |
| Myh1-F | GGCAGCAGCAGCTGCGGAAGCAGAGTCTGG | [[5](#_ENREF_5)] |
| Myh1-R | GAGTGCTCCTCAGATTGGTCATTAGC |  |
| Tnni2-F | GCACCTGAAGAGTGTGATGCT | This study |
| Tnni2-R | TCTCCTTCTCAGATTCTCGGC |  |
| Pgc1a-F | CCGTAAATCTGCGGGATGATG | [[6](#_ENREF_6)] |
| Pgc1a-R | CAGTTTCGTTCGACCTGCGTAA |  |
| Cpt1b-F | GAGACAGGACACTGTGTGGGTGA | [[6](#_ENREF_6)] |
| Cpt1b-R | AGTGCCTTGGCTACTTGGTACGAG |  |
| Acsl1-F | TTTGCCTGCAGCGAGTGTG | [[6](#_ENREF_6)] |
| Acsl1-R | GCCCTCGACTATCCCTATGGTAAGA |  |
| Fabp1-F | GGAATTGGGAGTAGGAAGAGCC | [[7](#_ENREF_7)] |
| Fabp1-R | TGGACTTGAACCAAGGAGTCAT |  |
| Acadl-F | GGACTCCGGTTCTGCTTCCA | [[6](#_ENREF_6)] |
| Acadl-R | TGCAATCGGGTACTCCCACA |  |
| Pfkl-F | CGTTGAGGTAGGAATACTTCTGCA | [[8](#_ENREF_8)] |
| Pfkl-R | ACCTCTTCCGAAAGGAGTGGA |  |
| Fasn-F | GGAGGTGGTGATAGCCGGTAT | [[9](#_ENREF_9)] |
| Fasn-R | TGGGTAATCCATAGAGCCCAG |  |
| Atgl-F | AACACCAGCATCCAGTTCAA | [[10](#_ENREF_10)] |
| Atgl-R | GGTTCAGTAGGCCATTCCTC |  |
| Scd1-F | TTCTTGCGATACACTCTGGTGC | [[9](#_ENREF_9)] |
| Scd1-R | CGGGATTGAATGTTCTTGTCGT |  |
| Cox2-F | CCGACTAAATCAAGCAACAGTAACA | [[11](#_ENREF_11)] |
| Cox2-R | AAATTTCAGAGCATTGGCCATAG |  |
| Cox4-F | CTATGTGTATGGCCCCATCC | [[11](#_ENREF_11)] |
| Cox4-R | AGCGGGCTCTCACTTCTTC |  |
| Tfam-F | GGAATGTGGAGCGTGCTAAAA | [[12](#_ENREF_12)] |
| Tfam-R | TGCTGGAAAAACACTTCGGAATA |  |
| Hk2-F | TGCTACAGGTCCGAGCCA | [[8](#_ENREF_8)] |
| Hk2-R | ATGCTGTCGTCACACGTGC |  |
| Cs-F | GGACAATTTTCCAACCAATCTGC | [[13](#_ENREF_13)] |
| Cs-R | TCGGTTCATTCCCTCTGCATA |  |
| Ppara-F | GACAAGGCCTCAGGGTACCA | [[14](#_ENREF_14)] |
| Ppara-R | GCCGAATAGTTCGCCGAAA |  |
| Ppia-F | CCTTGGGCCGCGTCTCCTT | [[15](#_ENREF_15)] |
| Ppia-R | CACCCTGGCACATGAATCCTG |  |
| Ucp2-F | GCAAGCATGTGTATGGCACAGTAAC | [[6](#_ENREF_6)] |
| Ucp2-R | AAATGTGGGCCTTCGGTCAG |  |

**Supporting References**

1. Koren L, Elhanani O, Kehat I, Hai T, Aronheim A. Adult cardiac expression of the activating transcription factor 3, ATF3, promotes ventricular hypertrophy. PLoS One. 2013;8(7):e68396. doi: 10.1371/journal.pone.0068396. PubMed PMID: 23874609; PubMed Central PMCID: PMCPMC3707568.

2. Gong SP, Lee EJ, Lee ST, Kim H, Lee SH, Han HJ, et al. Improved establishment of autologous stem cells derived from preantral follicle culture and oocyte parthenogenesis. Stem Cells Dev. 2008;17(4):695-712. doi: 10.1089/scd.2007.0168. PubMed PMID: 18752416.

3. Park KH, Franciosi S, Leavitt BR. Postnatal muscle modification by myogenic factors modulates neuropathology and survival in an ALS mouse model. Nat Commun. 2013;4:2906. doi: 10.1038/ncomms3906. PubMed PMID: 24346342; PubMed Central PMCID: PMCPMC4965267.

4. Liu J, Liang X, Zhou D, Lai L, Xiao L, Liu L, et al. Coupling of mitochondrial function and skeletal muscle fiber type by a miR-499/Fnip1/AMPK circuit. EMBO Mol Med. 2016. doi: 10.15252/emmm.201606372. PubMed PMID: 27506764.

5. Gan Z, Rumsey J, Hazen BC, Lai L, Leone TC, Vega RB, et al. Nuclear receptor/microRNA circuitry links muscle fiber type to energy metabolism. The Journal of clinical investigation. 2013;123(6):2564-75. Epub 2013/05/17. doi: 10.1172/JCI67652. PubMed PMID: 23676496; PubMed Central PMCID: PMC3668841.

6. Tian Z, Miyata K, Tabata M, Yano M, Tazume H, Aoi J, et al. Nifedipine increases energy expenditure by increasing PGC-1alpha expression in skeletal muscle. Hypertens Res. 2011;34(11):1221-7. doi: 10.1038/hr.2011.129. PubMed PMID: 21814214.

7. Nefzger CM, Jarde T, Rossello FJ, Horvay K, Knaupp AS, Powell DR, et al. A Versatile Strategy for Isolating a Highly Enriched Population of Intestinal Stem Cells. Stem Cell Reports. 2016;6(3):321-9. doi: 10.1016/j.stemcr.2016.01.014. PubMed PMID: 26923822; PubMed Central PMCID: PMCPMC4788784.

8. Din S, Konstandin MH, Johnson B, Emathinger J, Volkers M, Toko H, et al. Metabolic dysfunction consistent with premature aging results from deletion of Pim kinases. Circ Res. 2014;115(3):376-87. doi: 10.1161/CIRCRESAHA.115.304441. PubMed PMID: 24916111; PubMed Central PMCID: PMCPMC4254755.

9. Wu H, Gao Y, Shi HL, Qin LY, Huang F, Lan YY, et al. Astragaloside IV improves lipid metabolism in obese mice by alleviation of leptin resistance and regulation of thermogenic network. Sci Rep. 2016;6:30190. doi: 10.1038/srep30190. PubMed PMID: 27444146; PubMed Central PMCID: PMCPMC4957129.

10. Shen WJ, Patel S, Yu Z, Jue D, Kraemer FB. Effects of rosiglitazone and high fat diet on lipase/esterase expression in adipose tissue. Biochimica et biophysica acta. 2007;1771(2):177-84. doi: 10.1016/j.bbalip.2006.11.009. PubMed PMID: 17215164; PubMed Central PMCID: PMCPMC1933526.

11. Miura S, Kai Y, Kamei Y, Ezaki O. Isoform-specific increases in murine skeletal muscle peroxisome proliferator-activated receptor-gamma coactivator-1alpha (PGC-1alpha) mRNA in response to beta2-adrenergic receptor activation and exercise. Endocrinology. 2008;149(9):4527-33. doi: 10.1210/en.2008-0466. PubMed PMID: 18511502.

12. Aguilar V, Alliouachene S, Sotiropoulos A, Sobering A, Athea Y, Djouadi F, et al. S6 kinase deletion suppresses muscle growth adaptations to nutrient availability by activating AMP kinase. Cell Metab. 2007;5(6):476-87. doi: 10.1016/j.cmet.2007.05.006. PubMed PMID: 17550782.

13. Ho D, Zhao X, Yan L, Yuan C, Zong H, Vatner DE, et al. Adenylyl Cyclase Type 5 Deficiency Protects Against Diet-Induced Obesity and Insulin Resistance. Diabetes. 2015;64(7):2636-45. doi: 10.2337/db14-0494. PubMed PMID: 25732192; PubMed Central PMCID: PMCPMC4477357.

14. Waki H, Park KW, Mitro N, Pei L, Damoiseaux R, Wilpitz DC, et al. The small molecule harmine is an antidiabetic cell-type-specific regulator of PPARgamma expression. Cell Metab. 2007;5(5):357-70. Epub 2007/05/10. doi: S1550-4131(07)00073-3 [pii]10.1016/j.cmet.2007.03.010. PubMed PMID: 17488638.

15. Kreuzaler PA, Staniszewska AD, Li W, Omidvar N, Kedjouar B, Turkson J, et al. Stat3 controls lysosomal-mediated cell death in vivo. Nat Cell Biol. 2011;13(3):303-9. doi: 10.1038/ncb2171. PubMed PMID: 21336304.
