# Supplementary material for: Narciclasine attenuates diet-induced obesity by promoting oxidative metabolism in skeletal muscle
Source: PLoS Biol. 2017 Feb 16;15(2):e1002597. doi: 10.1371/journal.pbio.1002597 (PMC5331945; doi:10.1371/journal.pbio.1002597)
Supplement: S1 Text — (DOCX) [file pbio.1002597.s016.docx]

**Supporting Materials and Methods**

**Body Composition Measurement**

The fat and lean mass of each mouse in this study were measured weekly by an EchoMRI-100 body composition analyzer (Echo Medical Systems).

**MRI Acquisition**

The *in vivo* MRI experiments were conducted in compliance with and were approved by the local institutional committee. MRI measurements were performed on a 7 T Bruker ClinScan using a 40-mm volume and receiver coil. Prior to the *in vivo* experiments, the mice were initially anesthetized with 2% isoflurane in a dedicated chamber. During the course of the MRI experiments, anaesthesia levels were reduced to 1.5–1% in a combination of medical air and medical oxygen. Mice were positioned in the prone position, and respiratory-gated image acquisition was performed. Physiological monitoring was performed using an ML880 16/30 Powerlab system (AD Instruments, Spechbach, Germany). The temperature probe was placed inside the rectums of the mice, and the body temperature was monitored and controlled with circulating hot water. The imaging experiments were performed on NCD-veh, NCD-ncls, HFD-veh and HFD-ncls mice at 15 weeks of age. T2 weighted coronal images were obtained to localize the lumber I to lumber V abdomen regions using turbo spin echo imaging. Respiration-gated Dixon imaging was performed in the transverse plane by acquiring 22 1-mm-thick slices (time of repetition- 8 ms, number of averages- 2; echo time (opposite phase)- 1 ms; echo time (in phase)-2.5 ms; flip agnle-6º; echo band widths- 1090 and 1500 Hz/pixel; matrix size-192 x 256; and in-plane resolution-.0195 x 0.195).

**Image Processing**

The segmentation of subcutaneous adipose tissue (SAT) and visceral adipose tissue (VAT) was performed using a hybrid algorithm (active contour [[1](#_ENREF_1)] and fuzzy c-means –FCM [[2](#_ENREF_2)]) by an in-house-developed MATLAB program. Dixon-based fat images were first filtered using a bifilter to reduce the high frequency noise, to homogenize the regions with little intensity variation and to preserve and sharpen the edges. The denotations of the edges were improved using edge enhancement techniques. The initial curve was derived from the binary image of the data for each slice. Geodesic region- and curve evolution-based segmentation was used to identify the boundaries between the SAT and VAT. The initial contour for the algorithm was derived semi-automatically by selecting a few seed points near the abdominal wall. The geodesic curve was allowed to converge into the abdominal wall. After separation of the SAT and VAT regions, FCM with a different number of classes was performed on the SAT and VAT regions; then, thresholds for classifying the data into fat and non-fat regions were derived using statistical parameters such as the mean, variance and neighborhood relation. The different FCM regions in SAT and VAT were merged based on the intensity and neighborhood relation. The quantitation of the SAT and VAT regions was performed using the voxel dimensions of the scan.

**Histological Analysis**

Histological analysis was performed as previously described [[3](#_ENREF_3)]. Briefly, complete necropsies were performed, and the tissues used for histology were fixed in 10% neutral buffered formalin and embedded in paraffin, sectioned at 4 μm, stained with H&E, and examined for pathological findings.

**Tissue Lipid Content Measurement**

TG contents in BAT, liver and quadriceps skeletal muscle were measured using a Triglyceride Quantification Kit (Abcam). Up to 60 mg of each snap-frozen piece of tissue were homogenized in 5% NP-40 buffer and slowly heated to 90°C to solubilize all of the TGs. After centrifugation, the soluble fraction was assayed according to the manufacturer’s instructions and normalized to the tissue weight.

**Blood Metabolic Parameters**

Plasma insulin and leptin levels were measured after overnight fasting using the corresponding ELISA kits (Millipore). FFA, TG and cholesterol levels were measured with a commercial kit (WAKO) or with the Liquicor assay (Stanbio).

**Oral Glucose Tolerance Test**

All mice were fasted overnight. After baseline glucose values were measured using Accu-Check Performa (Roche), the mice were given glucose at 2 mg/g body weight by oral gavage. Subsequently, the clearance of plasma glucose was monitored at 15, 30, 60, 90 and 120 min following glucose administration.

**Glucose Stimulated Insulin Secretion Assay**

Insulin levels were determined using the Ultra Sensitive Mouse Insulin Immunoassay Kit (Antibody and Immunoassay Services, #32380) as per manufacturer’s instructions. Briefly, all mice were fasted for 16 hours, and then given glucose at 2 mg/g body weight by oral gavage. Insulin levels were determined from blood collected at 0, 8, 15, 30, and 60 min after glucose administration by measuring optical densities at 450 nm using a spectrometry plate reader (Tecan). Insulin concentration was calculated based on insulin standard using a 4 parameter logistic curve fitting program (GraphPad Software, Inc., San Diego, CA, USA).

**Insulin Tolerance Test**

All mice were fasted for 4 hours. After measuring blood glucose of each mouse using Accu-Check Performa (Roche) to get baseline data, the mice were given 0.75 U/kg body weight of insulin via IP injection. Subsequently, blood glucose levels were measured at 15, 30, 60, 90, and 120 min after insulin injection.

- **Whole Body Glucose and Lipid Oxidation Rates**
- Oxygen consumption (VO_2_) (L/min) and carbon dioxide production (VCO_2_) (L/min) were monitored by indirect calorimetry using the Oxymax/Comprehensive Lab Animal Monitoring System (Columbus Instrument, Ohio). Whole body substrate utilization rate were calculated using the equation used by Ferrannini [[4](#_ENREF_4)]: Glucose oxidation rate (g/min) = 4.55VCO_2_-3.21VO_2_-2.87N; Lipid oxidation rate (g/min) = 1.67(VO_2_-VCO_2_)-1.92N. Because the protein oxidation rate (N) (mg/min) contributes very little to the energy expenditure and the resting metabolic rate, we have ignored protein oxidation contribution as previously described [[5](#_ENREF_5)].

**Fecal Energy Output**

- Mice were individually housed for a period of 4 days during the last week of the study. Feces were collected every 24 hours and food debris was removed from the feces sample. Feces were then dried in an oven at 50°C. Energy content from 600 mg of dried feces was analyzed for each mouse using a bomb calorimeter (Parr Isoperibol Calorimeter, Model 6200, Parr Instrument Company).

**Open Field Test**

The open field test is commonly used to assess anxiety in rodents [[6](#_ENREF_6)]. The testing apparatus is a 50 × 50 cm open, grey, acrylic box (open field) with 20 cm high walls. Because rodents have an innate fear of open and bright spaces, they preferentially spend more time at the perimeter rather than the centre of the open field. The time spent in the centre versus the perimeter is taken as a measure of anxiety-like behaviour. Test sessions lasted 10 min and were video recorded. The number of entries into the centre and the time spent in the centre versus perimeter were scored using an automated video-based tracking system (Noldus EthoVision 9, Netherlands).

**Elevated Plus Maze Test**

The elevated plus maze [[7](#_ENREF_7)] is a well-established test of anxiety. The testing apparatus is shaped like a ‘+’ with two open arms perpendicular to two closed arms of equal dimensions. The closed arms are enclosed by three 10-cm high walls. Because rodents have an innate fear of elevated open spaces, they tend to spend less time in the open arms. Time spent in the open versus closed arms is taken as a measure of anxiety-like behaviour. Test sessions lasted 5 min and were video recorded. The number of entries into the open arms and time spent in the open versus closed arms were scored manually.

**Immunofluorescence**

Serial sections of quadriceps muscle were cut at 4 µm thicknesses, deparaffinized, and rehydrated with xylenes, ethanol, and water by standard methods. Tissue sections were incubated for 5 minutes in 0.3% Triton X100-PBS, and blocked for 1 hour in 5% normal donkey serum-PBS and then incubated overnight with anti-Myh7 antibody (Santa Cruz) diluted 1:150 in 1% BSA - 0.1% Triton X100-PBS at 4°C. The tissue sections were then washed 3 times with PBS for 10 minutes before incubation with Alexa Fluor 388 donkey anti-mouse (Invitrogen) (0.2 U/ml) and mounted. Samples were examined using Eclipse Ti Nikon confocal laser scanning microscope.

**Muscle Fiber Typing**

Skeletal muscle fiber typing in quadriceps was performed using immunofluorescence staining against myosin heavy chain proteins MHC I, MHC IIa and MHC IIb. Briefly, serial cryosections of quadriceps muscle were immunostained with specific MHC antibodies from Developmental Studies Hybridoma Bank (DSHB), The University of Iowa: (1) anti-MHC I (A4.840, 1:50) [[8](#_ENREF_8), [9](#_ENREF_9)]; (2) anti-MHC IIa (2F7, 1:70) [[10](#_ENREF_10)]; (3) anti-MHC IIb (10F5, 1:20) [[10](#_ENREF_10), [11](#_ENREF_11)], then incubated with fluorescent secondary antibody (with conjugated Alexa 488). Stained sections were examined under the fluorescence microscope (Leica) and the percentage of each fiber type (MHC I, MHC IIa and MHC IIb) was determined by manual counting of immuno-positive fibers from each section. The cross-sectional area of muscle fibers were quantified from H&E stained muscle sections using ImageJ.

- **Grip Strength Test**
- Grip strength of both forelimb and hindlimb (4 paws) was measured using a MK-380M grip strength meter (Muromachi Kikai Co., Ltd). The grip strength of each mouse from each group was measured 3 times with a 3 min interval between each measurement to prevent fatigue of animals. Measurements were performed before and after ncls treatment. Individual muscular function was assessed by sensing the peak amount of force for each measurement.
- **Muscle Mass**
- At the end of the study, mice were anaesthetized and quadriceps, tibialis anterior, gastrocnemius, EDL and soleus muscles were dissected and cleaned from visible fat deposits. Each muscle was individually weighted on a Precisions’ weight balance. Mass of individual dissected muscle were compiled and presented as box and whisker plots.

**Core Body Temperature**

The core body temperature was measured with a probe thermometer (Advance Technology) at a constant depth as described previously [[12](#_ENREF_12)].

**Mitochondrial DNA Content**

Mitochondrial DNA copy numbers were determined by semi-quantitative real-time PCR analysis of total DNA isolated from muscle, BAT, liver and WAT of HFD-veh and HFD-ncls mice. Primer sequences for mitochondrial DNA (mtCox1) and nuclear DNA (PECAM) were from [[13](#_ENREF_13)].

**Immunoblotting**

Muscle, liver, BAT and WAT tissues were harvested and homogenized in PLC lysis buffer (50 mM HEPES, pH=7.5; 150 mM NaCl; 10% glycerol; 1 mM EGTA; 1% triton-100) containing protease inhibitor cocktail and phosphatase inhibitor cocktail (Roche). Homogenized tissues were kept on ice for 30 min with vigorous vortexing every 5 min, centrifuged at 12,000 rpm for 15 min at 4°C. The supernatant was collected for immunoblotting analysis. Before western blot analysis, protein concentration of the whole-cell lysates and tissue extracts was determined by the bicinchoninic acid assay (Sigma). Equal amount of lysates from C2C12 myotubes or mouse tissues were resolved by SDS-PAGE, transferred onto PVDF membranes, and probed with the Phospho-AMPKα (Thr172) (40H9) Rabbit mAb (#2535, Cell Signaling), AMPKα (D5A2) Rabbit mAb (#5831, Cell Signaling), Phospho-Acetyl-CoA Carboxylase (Ser79) (D7D11) Rabbit mAb (#11818, Cell Signaling), Acetyl-CoA Carboxylase (C83B10) Rabbit mAb (#3676, Cell Signaling), UCP2 (G6) Mouse mAb (sc-390189, Santa Cruz) and an antibody against GAPDH (sc-32233, Santa Cruz). The intensities of the bands of interest were determined by the Quantity One 1-D analysis software (BIO-RAD).

**Cell Staining**

- Differentiated murine and human myotubes were fixed in 4% paraformaldehyde/phosphate-buffered saline (PBS) for 10 min at 25°C. Following fixation, the samples were washed 3 times in PBS for 10 min. After washing, fixed myotubes were stained with 1 μg/ml BODIPY 493/503 dye (Molecular Probes) in 150 mM NaCl-water for 10 min at 25°C. Sections were then mounted with Prolong Gold Antifade with DAPI (Molecular Probes) and covered with glass coverslips. Images were acquired using an Eclipse Ti Nikon confocal laser scanning microscope.

**Supporting References**

1. Lankton S, Tannenbaum A. Localizing region-based active contours. IEEE transactions on image processing : a publication of the IEEE Signal Processing Society. 2008;17(11):2029-39. Epub 2008/10/16. doi: 10.1109/TIP.2008.2004611. PubMed PMID: 18854247; PubMed Central PMCID: PMC2796112.

2. Bezdek JC. Pattern Recognition with Fuzzy Objective Function Algorithms. Plenum Press, New York. 1981.

3. Julien SG, Dube N, Read M, Penney J, Paquet M, Han Y, et al. Protein tyrosine phosphatase 1B deficiency or inhibition delays ErbB2-induced mammary tumorigenesis and protects from lung metastasis. Nature genetics. 2007;39(3):338-46. Epub 2007/01/30. doi: 10.1038/ng1963. PubMed PMID: 17259984.

4. Ferrannini E. The theoretical bases of indirect calorimetry: a review. Metabolism: clinical and experimental. 1988;37(3):287-301. Epub 1988/03/01. PubMed PMID: 3278194.

5. Tadaishi M, Miura S, Kai Y, Kano Y, Oishi Y, Ezaki O. Skeletal muscle-specific expression of PGC-1alpha-b, an exercise-responsive isoform, increases exercise capacity and peak oxygen uptake. PloS one. 2011;6(12):e28290. Epub 2011/12/17. doi: 10.1371/journal.pone.0028290. PubMed PMID: 22174785; PubMed Central PMCID: PMC3234261.

6. Crawley JN. Exploratory behavior models of anxiety in mice. Neurosci Biobehav Rev. 1985;9(1):37-44. PubMed PMID: 2858080.

7. Walf AA, Frye CA. The use of the elevated plus maze as an assay of anxiety-related behavior in rodents. Nature protocols. 2007;2(2):322-8. doi: 10.1038/nprot.2007.44. PubMed PMID: 17406592; PubMed Central PMCID: PMCPMC3623971.

8. Giorgetti E, Yu Z, Chua JP, Shimamura R, Zhao L, Zhu F, et al. Rescue of Metabolic Alterations in AR113Q Skeletal Muscle by Peripheral Androgen Receptor Gene Silencing. Cell Rep. 2016;17(1):125-36. doi: 10.1016/j.celrep.2016.08.084. PubMed PMID: 27681426.

9. Webster C, Silberstein L, Hays AP, Blau HM. Fast muscle fibers are preferentially affected in Duchenne muscular dystrophy. Cell. 1988;52(4):503-13. PubMed PMID: 3342447.

10. Lucas CA, Kang LH, Hoh JF. Monospecific antibodies against the three mammalian fast limb myosin heavy chains. Biochem Biophys Res Commun. 2000;272(1):303-8. doi: 10.1006/bbrc.2000.2768. PubMed PMID: 10872844.

11. Thomas MM, Wang DC, D'Souza DM, Krause MP, Layne AS, Criswell DS, et al. Muscle-specific AMPK beta1beta2-null mice display a myopathy due to loss of capillary density in nonpostural muscles. FASEB journal : official publication of the Federation of American Societies for Experimental Biology. 2014;28(5):2098-107. doi: 10.1096/fj.13-238972. PubMed PMID: 24522207; PubMed Central PMCID: PMCPMC3986832.

12. Kim HJ, Cho H, Alexander R, Patterson HC, Gu M, Lo KA, et al. MicroRNAs are required for the feature maintenance and differentiation of brown adipocytes. Diabetes. 2014;63(12):4045-56. doi: 10.2337/db14-0466. PubMed PMID: 25008181; PubMed Central PMCID: PMCPMC4238002.

13. Zhao XY, Li S, Wang GX, Yu Q, Lin JD. A long noncoding RNA transcriptional regulatory circuit drives thermogenic adipocyte differentiation. Mol Cell. 2014;55(3):372-82. doi: 10.1016/j.molcel.2014.06.004. PubMed PMID: 25002143; PubMed Central PMCID: PMC4127104.
